# Novel Antimicrobial and Antitumor Agents Bearing Pyridine-1,2,4-triazole-3-thione-hydrazone Scaffold: Synthesis, Biological Evaluation, and Molecular Docking Investigation

**DOI:** 10.3390/biom14121529

**Published:** 2024-11-28

**Authors:** Aida Šermukšnytė, Maryna Stasevych, Olena Komarovska-Porokhnyavets, Viktor Zvarych, Eglė Jakubauskienė, Kristina Kantminienė, Ingrida Tumosienė

**Affiliations:** 1 Department of Organic Chemistry, Kaunas University of Technology, Radvilėnų pl. 19, 50254 Kaunas, Lithuania; aida.sermuksnyte@ktu.edu (A.Š.); ingrida.tumosiene@ktu.lt (I.T.); 2 Department of Technology of Biologically Active Substances, Pharmacy, and Biotechnology, Lviv Polytechnic National University, S. Bandera Str. 12, 79013 Lviv, Ukraine; maryna.v.stasevych@gmail.com (M.S.); olena.z.komarovska-porokhniavets@lpnu.ua (O.K.-P.); 3 Department of Automated Control Systems, Lviv Polytechnic National University, S. Bandera Str. 12, 79013 Lviv, Ukraine; viktor.i.zvarych@lpnu.ua; 4 Institute of Biotechnology, Life Sciences Center, Vilnius University, Saulėtekio al. 7, 10257 Vilnius, Lithuania; egle.jakubauskiene@bti.vu.lt; 5 Department of Physical and Inorganic Chemistry, Kaunas University of Technology, Radvilėnų pl. 19, 50254 Kaunas, Lithuania

**Keywords:** pyrrolidin-2-one, hydrazone, triple-negative breast cancer, glioblastoma, cell viability, MTT assay, molecular modeling

## Abstract

A series of target 4-substituted-5-(2-(pyridine-2-ylamino)ethyl)-2,4-dihydro-3*H*-1,2,4-triazole-3-thiones and their chloro analogs **7**–**21** were synthesized in a reaction of the selected aldehydes with the corresponding 4-amino-1,2,4-triazole-3-thiones **5** and **6**, which were obtained from 3-(pyridin-2-ylamino)propanoic acid (**3**) or 3-((5-chloropyridin-2-yl)amino)propanoic acid (**4**), respectively, with thioacetohydrazide. The antibacterial and antifungal activities of the synthesized hydrazones were screened against the bacteria *Escherichia coli*, *Staphylococcus aureus*, and *Mycobacterium luteum* and the fungi *Candida tenuis* and *Aspergillus niger* by agar diffusion and serial dilution methods. 4-Amino-5-(2-((5-chloropyridin-2-yl)amino)ethyl)-2,4-dihydro-3*H*-1,2,4-triazole-3-thione (**6**) and 4-(benzylideneamino)-5-(2-(pyridin-2-ylamino)ethyl)-2,4-dihydro-3*H*-1,2,4-triazole-3-thione (**7**) were identified as exceptionally active (MIC 0.9 µg/mL) against the fungus *C. tenuis.* 5-Chloropyridine derivatives bearing 4-benzylidene **8**, 2-nitrobenzylidene **10**, pyridinylmethylene **16**, and 4-methylthiobenzylidene **21** moieties showed very high antibacterial activity (MIC 3.9 µg/mL) against the *M. luteum* strain. The cell viability screening of the synthesized compounds using triple-negative breast cancer MDA-MB-231 and glioblastoma U-87 cell lines by MTT assay identified three active hydrazones, of which 5-(2-(pyridin-2-ylamino)ethyl)-4-((pyridin-3-ylmethylene)amino)-2,4-dihydro-3*H*-1,2,4-triazole-3-thione (**15**) had the highest effect on the viability of cells (IC_50_ value 39.2 ± 1.7 μM against MDA-MD-231). The in silico molecular modeling results suggested that these three most active hydrazones might have influenced the mitogen-activated protein kinase pathway through the inhibition of BRAF and MEK serine–threonine protein kinases. 5-(2-((5-Chloropyridin-2-yl)amino)ethyl)-4-((4-(methylthio)benzylidene)amino)-2,4-dihydro-3*H*-1,2,4-triazole-3-thione (**21**) demonstrated the highest affinity among them.

## 1. Introduction

Antimicrobial resistance (AMR) poses a significant and ever-growing global threat to public health, economic stability, and clinical practice [1]. In 2019, an estimated 4.95 million deaths were associated with bacterial AMR evaluated for 23 pathogens and 88 pathogen–drug combinations [2].

The ability of microorganisms, including bacteria, viruses, fungi, and parasites, to withstand the action of antimicrobial drugs designed to kill them or inhibit their growth has led to the resurgence of infectious diseases that were once thought to be under control [3]. AMR not only complicates treatment but also leads to longer hospital stays, higher medical costs, and increased mortality rates. Traditional antibiotics are increasingly ineffective; therefore, the development of novel therapeutic strategies and new antimicrobial agents is one of the ways out of this situation, as has been indicated by the World Health Organization (WHO).

Another major life-threatening disease and a considerable barrier to increasing life expectancy worldwide is cancer [4]. It is a leading cause of death worldwide, accounting for approx. 9.7 million deaths in 2022 alongside 20 million new cases of cancer [5]. By 2040, the global cancer burden is expected to increase by 47% in comparison to 2020 [6]. Despite summoned global efforts in cancer research and remarkable results for some types of cancer, the main goal of a durable cure remains unachieved for the majority of cancer types [7]. Therefore, the search for innovative therapeutic solutions and efficient anticancer agents is of high importance.

In the ongoing attempts to address both of the global health challenges discussed above, recent advances in synthetic chemistry and molecular design have paved the way for a different approach, i.e., the creation of hybrid compounds that comprise functional moieties, enabling interaction with several specific biological targets, and thus exhibit both antimicrobial and anticancer properties simultaneously. Hybrid compounds possess potential advantages over mixtures used in combination therapies [8]. Antimicrobial and anticancer drugs share common targets and mechanisms of action [9]. According to research findings, antibiotics can promote cancer apoptosis, inhibit cancer growth, and prevent cancer metastasis. For these reasons, antibiotics are increasingly used to assist in the treatment of cancers [10].

In pharmaceutical targets, pyridine and its precursor molecule dihydropyridine are among the most prevalent structural units. Pyridine- and dihydropyridine-containing drugs constitute nearly 14% and 4%, respectively, of *N*-heterocyclic drugs approved by the US Food and Drug Administration (FDA). The major therapeutic areas of focus of 18% of these drugs are infectious diseases, inflammation, the nervous system, and oncology [11]. The chemotherapeutic agent crizotinib, containing pyridine skeleton as part of its chemical structure, is used for the treatment of non-small cell lung cancer. It acts as an anaplastic lymphoma kinase (ALK) and ROS1 (c-ros oncogene1) inhibitor. Numerous pyridine-based compounds have been identified as PIM-1 kinase inhibitors, human carbonic anhydrase inhibitors, proto-oncogene tyrosine-protein kinase (ROS) inhibitors, ALK/ROS1 dual inhibitors, receptor tyrosine kinase (RTK) c-Met inhibitors, epidermal growth factor receptors, EGFR and HER-2 kinase inhibitors, cyclin-dependent kinase (CDK) inhibitors, VEGFR-2 inhibitors, topoisomerases, phosphoinositide 3-kinase inhibitors, maternal embryonic leucine zipper kinase (MELK) inhibitors, NF-κB inhibitors, etc. [12]. 2-(4-(2-(Dimethylamino)ethyl)-4*H*-1,2,4-triazole-3-pyridine derivatives have been reported to possess in vitro cytotoxic activity against MKN-45, H460, HT-29, A549, and U87MG cancer cells [13].

Sulfapyridine is a first-generation sulfonamide antibiotic. It is no longer prescribed for the treatment of infections in humans; however, it is used in veterinary medicine. Numerous compounds containing the pyridine nucleus, along with other heterocycles or fused with other heterocycles, have been reported to possess antimicrobial activity against different strains including *S. aureus*, *B. subtilis*, *E. coli*, *C. albicans*, and *A. niger* [14].

The presence of the chlorine atom in the structure of various (or different) compounds has been shown to modulate biological activity by changing its lipophilicity and, therefore, influencing its permeability through membranes and penetration into cells [15]. D. Nawrot et al. reported a comprehensive study of the structure–activity relationship of 44 differently substituted *N*-pyridinylbenzamides and demonstrated that the presence of a chlorine substituent in the pyridine ring enhances the antimycobacterial activity of the synthesized compounds [16]. As reported by S. Cyboran-Mikołajczyk et al., the presence of a chloro substituent in the structure of synthesized 2′-hydroxychalcone molecules improved their antimicrobial and antiproliferative effect, especially against aggressive breast cancer cell lines [15].

1,2,4-Triazole-based derivatives have driven the interest of medicinal chemists because of their fascinating pharmacophoric features. The electron-rich nature of 1,2,4-triazoles helps in binding with various biological targets and enzymes and, subsequently, exhibits a broad range of biological activities including those that are antibacterial, antifungal, antitumor, anti-inflammatory, antitubercular, hypoglycaemic, antidepressant, anticonvulsant, analgesic, antiviral, anticancer, antimalarial, antioxidant, etc. [17]. Among 1,2,4-triazole derivatives, mercapto- and thione-substituted 1,2,4-triazole ring systems have been well studied and, so far, a variety of biological activities have been reported for a large number of their derivatives, such as antibacterial, antifungal, antitubercular, antimycobacterial, anticancer, diuretic, and hypoglycemic properties [18]. Furthermore, the triazole ring may be utilized as a binding core in forming bi-functional drug molecules when combining different pharmacophore moieties [19].

Since pyridine and 1,2,4-triazole moieties have individually demonstrated notable pharmacological activities, including anticancer and antimicrobial effects, merging these structural units into a hybrid framework may utilize the potential synergies leading to compounds with superior dual functionality. Based on the results of our previous studies of the structure–activity relationship of hydrazones comprising different heterocyclic moieties [20,21,22] and our search for heterocyclic compounds possessing antimicrobial properties [23,24,25], we report the synthesis of a series of hydrazones bearing pyridine and 1,2,4-triazole-3-thione moieties **7**–**21** and the in vitro evaluation of their antibacterial activity against *Escherichia coli*, *Staphylococcus aureus*, and *Mycobacterium luteum* and their antifungal activity against *Candida tenuis* and *Aspergillus niger* by agar diffusion and serial dilution methods. In vitro cytotoxicity screening of the synthesized compounds by MTT assay using triple-negative breast cancer MDA-MB-231 and glioblastoma U-87 cell lines provided valuable information about the viability and proliferation of the cancer cells of choice in the presence of potential anticancer agents. In silico molecular modeling results revealed that compounds **15**, **16**, and **21**, possessing the highest in vitro anticancer activity, are potential kinase inhibitors, probably acting on the mitogen-activated protein kinase pathway through the inhibition of BRAF and MEK serine–threonine protein kinases.

## 2. Materials and Methods

### 2.1. Chemistry

#### 2.1.1. Reagents and Instruments

Reagents were purchased from Sigma-Aldrich (St. Louis, MO, USA) and TCI Europe N.V. (Zwijndrecht, Belgium). The reaction course and purity of the synthesized compounds were monitored by TLC using aluminum plates precoated with silica gel 60 F254 (MerckKGaA, Darmstadt, Germany). The melting points were determined using MEL-TEMP melting point apparatus (Bibby Scientific Company, Burlington, NJ, USA) and were uncorrected. FT-IR spectra (ν, cm^−1^) were recorded using a PerkinElmer Spectrum BX FT–IR spectrometer (Perkin-Elmer Inc., Waltham, MA, USA) using KBr pellets. The ^1^H and ^13^C-NMR spectra were recorded in DMSO-*d*_6_ using a Bruker Avance III (400 MHz, 101 MHz) spectrometer (Bruker BioSpin AG, Fällanden, Switzerland) operating in the Fourier transform mode. Chemical shifts (*δ*) were reported in parts per million (ppm), calibrated from TMS (0 ppm) as an internal standard for ^1^H NMR and from DMSO-*d*_6_ (39.43 ppm) for ^13^C NMR. High-resolution mass spectra (HRMS) were obtained using a Bruker maXis UHR-TOF mass spectrometer (Bruker Daltonics, Bremen, Germany) with ESI ionization. 3-(Pyridin-2-ylamino)propanoic acid (**3**) and 3-((5-chloropyridin-2-yl)amino)propanoic acid (**4**) were synthesized as described in reference [26].

#### 2.1.2. 4-Amino-5-(2-(pyridin-2-ylamino)ethyl)-2,4-dihydro-3*H*-1,2,4-triazole-3-thione (**5**)

3-(Pyridin-2-ylamino)propanoic acid (**3**) (33.24 g, 0.20 mol) was melted with thiocarbohydrazide (21.23 g, 0.20 mol) at 200 °C for 3 h. Distilled water (100 mL) was added to the hot reaction mixture. The precipitate was collected by filtration and recrystallized from ethanol. Yield 34% (17.01 g), brown crystals, m.p. 214–215 °C. IR (KBr) ν_max_ (cm^−1^): 3295; 3243; 3082 (NH, NH_2_); 1234 (C=S). ^1^H NMR (400 MHz, DMSO-*d*_6_): *δ* = 2.89 (t; 2H; *J* = 6.80 Hz; H_8_), 3.59 (q; 2H; *J* = 13.2 Hz; *J* = 12.8 Hz; H_7_), 5.53 (s, 2H, NH_2_), 6.44–6.48 (m, 2H, H_6+_NH), 6.60 (t; H; *J* = 5.6 Hz; H_4_), 7.34 (t; H; *J* = 8.80 Hz; H_5_), 7.96 (d, H, H_3_), 13.47 (s, H, NH). ^13^C NMR (101 MHz, DMSO-*d*_6_): *δ* = 25.0 (C_8_), 37.4 (C_7_), 108.3, 111.7, 136.6, 147.5, 150.6, 158.5 (C_1,3,4,5,6,9_), 165.8 (C_10_). HRMS (ESI+): *m*/*z* calculated for C_9_H_12_N_6_S, 237.0923 [M+H]^+^; found, 237.0926.

#### 2.1.3. 4-Amino-5-(2-((5-chloropyridin-2-yl)amino)ethyl)-2,4-dihydro-3*H*-1,2,4-triazole-3-thione (**6**)

3-[(5-Chloropyridin-2-yl)amino]propanoic acid (**4**) (6.00 g, 0.07 mol) was melted with thiocarbohydrazide (9.56 g, 0.09 mol) at 200 °C for 2 h. Distilled water (80 mL) was added to the hot reaction mixture. The precipitate was collected by filtration and recrystallized from ethanol. Yield 74% (5.96 g), light brown crystals, m.p. 146–147 °C. IR (KBr) ν_max_ (cm^−1^): 1317 (C=S); 3055; 3231; 3273 (NH, NH_2_). ^1^H NMR (400 MHz, DMSO-*d*_6_): *δ* = 2.87 (t; 2H; *J* = 6.80 Hz; H_8_), 3.59 (q; 2H; *J* = 13.2 Hz; *J* = 12.8 Hz; H_7_), 5.51 (s, 2H, NH_2_), 6.49 (d; H; *J* = 6.80 Hz; H_6_), 6.89 (t; H; *J* = 6.00 Hz; NH), 7.41 (d; H; *J* = 8.80 Hz; H_5_), 7.95 (d, H, H_3_), 13.45 (s, H, NH). ^13^C NMR (101 MHz, DMSO-*d_6_*): *δ* = 24.8 (C_8_), 37.5 (C_7_), 109.8, 117.5, 136.5, 145.4, 150.5, 157.1 (C_1,3,4,5,6,9_), 165.9 (C_10_). HRMS (ESI+): *m*/*z* calculated for C_9_H_11_ClN_6_S, 271.0533 [M+H]^+^; found, 271.0531.

#### 2.1.4. General Procedure for Synthesis of Compounds **7**–**21**

1,2,4-Triazolethione (**5** or **6**) (1.5 mmol) was dissolved in methanol (30 mL) and the corresponding aldehyde (2.0 mmol) dissolved in methanol (10 mL) was added. The reaction mixture was stirred under reflux conditions for 5–18 h in the presence of a few drops of HCl. After the completion of the reaction, aqueous sodium acetate solution was added until the full precipitation of the product occurred. The precipitate was collected by filtration and recrystallized from a DMF/H_2_O mixture.

##### 4-(Benzylideneamino)-5-(2-(pyridin-2-ylamino)ethyl)-2,4-dihydro-3*H*-1,2,4-triazole-3-thione (**7**)

Prepared from benzaldehyde. Yield 65% (0.31 g), yellowish brown crystals, m.p. 204–205 °C. IR (KBr) ν_max_ (cm^−1^): 1222 (C=S); 3134; 3217 (NH). ^1^H NMR (400 MHz, DMSO-*d*_6_): *δ* = 3.00 (t; 2H; *J* = 6.6 Hz; H_8_), 3.61 (q; 2H; *J* = 12.8 Hz; *J* = 6.6 Hz; H_7_), 6.42–6.45 (m, H, H_4_), 6.70 (t; H; *J* = 4 Hz; NH), 7.27–7.31 (m, H, H_5_), 7.45–7.63 (m, 5H, H_13,14,15,16,17_), 7.87 (d, 2H, H_3,6_), 9.95 (s, H, H_11_), 13.78 (s, H, NH). ^13^C NMR (101 MHz, DMSO-*d*_6_): *δ* = 25.3 (C_8_), 37.7 (C_7_), 108.4, 111.7, 128.5, 128.8, 129.1, 130.1, 132.2, 132.6, 136.6, 147.3, 149.9, 158.3, 161.2 (C_1,3,4,5,6,9,11,12,13,14,15,16,17_), 163.2 (C_10_). HRMS (ESI+): *m*/*z* calculated for C_16_H_16_N_6_S, 325.1236 [M+H]^+^; found, 325.1233.

##### 4-(Benzylideneamino)-5-(2-((5-chloropyridin-2-yl)amino)ethyl)-2,4-dihydro-3*H*-1,2,4-triazole-3-thione (**8**)

Prepared from benzaldehyde. Yield 55% (0.27 g), grey crystals, m.p. 163–164 °C. IR (KBr) ν_max_ (cm^−1^): 1284 (C=S); 2946; 3099 (NH). ^1^H NMR (400 MHz, DMSO-*d*_6_): *δ* = 2.86 (t; 2H; *J* = 6.8 Hz; H_8_), 3.58 (t; 2H; *J* = 6.8 Hz; H_7_), 5.48 (s, H, NH), 6.49 (d; H; *J* = 6.8 Hz; H_6_), 6.88 (d; H; *J* = 6.8 Hz; H_5_), 7.80–7.87 (m, 5H, H_13,14,15,16,17_), 8.00 (s, 2H, H_3,11_), 11.41 (s, H, NH). ^13^C NMR (101 MHz, DMSO-*d*_6_): *δ* = 25.0 (C_8_), 37.7 (C_7_), 110.0, 117.6, 127.5, 128.8, 129.9, 134.4, 136.7, 142.4, 150.6, 157.3, 165.9 (C_1,3,4,5,6,9,11,13,14,15,16,17_), 175.9 (C_10_). HRMS (ESI+): *m*/*z* calculated for C_16_H_15_ClN_6_S, 359.0846 [M+H]^+^; found, 359.0844.

##### 4-((2-Nitrobenzylidene)amino)-5-(2-(pyridin-2-ylamino)ethyl)-2,4-dihydro-3*H*-1,2,4-triazole-3-thione (**9**)

Prepared from nitrobenzaldehyde. Yield 53% (0.29 g), yellow crystals, m.p. 207–208 °C. IR (KBr) ν_max_ (cm^−1^): 1244 (C=S); 3111; 3269 (NH). ^1^H NMR (400 MHz, DMSO-*d*_6_): *δ* = 3.00 (t; 2H; *J* = 6.6 Hz; H_8_), 3.60 (q; 2H; *J* = 12.8 Hz; *J* = 6.6 Hz; H_7_), 6.39 (t; H; *J* = 4 Hz; H_4_), 6.65 (t; H; *J* = 6.0 Hz; NH), 7.26 (t; H; *J* = 6.6 Hz; H_5_), 7.68 (t; H; *J* = 7.8 Hz; H_15_), 7.84–7.88 (m, 3H, H_6,14,16_), 7.91 (d, H, H_3_), 8.07 (s, 0.5H, H_11_), 8.08 (s, 0.5H, H_11_), 8.11–8.14 (m, H, H_13_), 11.08 (s, H, NH). ^13^C NMR (101 MHz, DMSO-*d*_6_): *δ* = 25.2 (C_8_), 38.4 (C_7_), 108.3, 111.6, 124.6, 124.7, 127.3, 128.4, 128.7, 130.7, 132.5, 133.6, 133.9, 136.5, 147.4, 148.3, 148.7, 149.9, 150.3, 158.4, 161.2 (C_1,3,4,5,6,9,11,13,14,15,16,17_), 175.9 (C_10_). HRMS (ESI+): *m*/*z* calculated for C_16_H_15_N_7_O_2_S, 370.1087 [M+H]^+^; found, 370.1097.

##### 5-(2-((5-Chloropyridin-2-yl)amino)ethyl)-4-((2-nitrobenzylidene)amino)-2,4-dihydro-3*H*-1,2,4-triazole-3-thione (**10**)

Prepared from nitrobenzaldehyde. Yield 74% (0.61 g), brown crystals, m.p. 195–196 °C. IR (KBr) ν_max_ (cm^−1^): 1271 (C=S); 3018; 3340 (NH). ^1^H NMR (400 MHz, DMSO-*d*_6_): *δ* = 3.03 (t; 2H; *J* = 6.6 Hz; H_8_), 3.59 (q; 2H; *J* = 12.8 Hz; *J* = 6.6 Hz; H_7_), 6.38 (d; H; *J* = 8.8 Hz; H_6_), 6.94 (t; H; *J* = 6.0 Hz; NH), 7.25 (d; H; *J* = 8.8 Hz; H_5_), 7.66–7.70 (m, 3H, H_14_,_15,16_), 8.03–8.08 (m, 2H, H_3,17_), 10.87 (s, H, H_11_), 13.86 (s, H, NH). ^13^C NMR (101 MHz, DMSO-*d*_6_): *δ* = 24.8 (C_8_), 38.1 (C_7_), 109.8, 117.5, 124.7, 124.8, 126.8, 128.6, 130.7, 132.8, 133.6, 133.9, 136.3, 145.3, 148.3, 148.7, 150.5, 155.5, 157.0, 161.3 (C_1,3,4,5,6,9,11,13,14,15,16,17_), 175.8 (C_10_). HRMS (ESI+): *m*/*z* calculated for C_16_H_14_ClN_7_O_2_S, 404.0697 [M+H]^+^; found, 404.0698.

##### 4-((4-(Dimethylamino)benzylidene)amino)-5-(2-(pyridin-2-ylamino)ethyl)-2,4-dihydro-3*H*-1,2,4-triazole-3-thione (**11**)

Prepared from 4-(dimethylamino)benzaldehyde. Yield 76% (0.41 g), reddish crystals, m.p. 274–275 °C. IR (KBr) ν_max_ (cm^−1^): 1228 (C=S); 3240 (NH). ^1^H NMR (400 MHz, DMSO-*d*_6_): *δ* = 2.93 (t; 2H; *J* = 6.6 Hz; H_8_), 3.02 (s, 6H, H_18,19_), 3.58 (q; 2H; *J* = 12.8 Hz; *J* = 6.6 Hz; H_7_), 6.39–6.46 (m, 2H, H_4,6_), 6.64 (t; H; *J* = 6.4 Hz; NH), 6.79 (d; 2H; *J* = 8.8 Hz; H_13,17_), 7.30 (t; H; *J* = 6.6 Hz; H_5_), 7.67 (d; 2H; *J* = 8.8 Hz; H_14,16_), 7.93 (d; H; *J* = 4.8 Hz; H_3_), 8.63 (s, H, H_11_), 9.45 (s, H, NH). ^13^C NMR (101 MHz, DMSO-*d*_6_): *δ* = 25.3 (C_8_), 37.6 (C_7_), 39.7 (C_18,19_), 108.3, 111.5, 111.6, 118.9, 129.5, 136.5, 147.5, 147.5, 149.3, 153.1, 158.4, 161.2 (C_1,3,4,5,6,9,11,12,13,14,15,16,17_), 164.6 (C_10_). HRMS (ESI+): *m*/*z* calculated for C_18_H_21_N_7_S, 368.1658 [M+H]^+^; found, 368.1656.

##### 5-(2-((5-Chloropyridin-2-yl)amino)ethyl)-4-((4-(dimethylamino)benzylidene)amino)-2,4-dihydro-3*H*-1,2,4-triazole-3-thione (**12**)

Prepared from 4-(dimethylamino)benzaldehyde. Yield 85% (0.68 g), dark green crystals. m.p. 170–171 °C. IR (KBr) ν_max_ (cm^−1^): 1304 (C=S), 2895, 3260 (NH). ^1^H NMR (400 MHz, DMSO-*d*_6_): *δ* = 2.92–2.97 (m, 2H, H_8_), 3.02 (s, 6H, H_18,19_), 3.57 (q; 2H; *J* = 6.4 Hz; *J* = 12.8 Hz; H_7_), 6.41 (d; H; *J* = 8.8 Hz; H_6_), 6.73–6.78 (m, 2H, H_13,17_), 6.92 (t; H; *J* = 8.8 Hz; NH), 7.32 (d; H; *J* = 8.8 Hz; H_5_), 7.60–7.65 (m, 3H, H_3,14,16_), 8.42 (s, H, H_11_), 11.47 (s, H, NH). ^13^C NMR (101 MHz, DMSO-*d*_6_): *δ* = 25.0 (C_8_), 37.9 (C_7_), 39.8 (C_18,19_), 109.8, 111.5, 111.7, 117.5, 118.7, 128.9, 130.3, 136.4, 145.3, 149.4, 151.5, 153.1, 157.0, 161.2, 165.0 (C_1,3,4,5,6,9,11,13,14,15,16,17_), 173.2 (C_10_). HRMS (ESI+): *m*/*z* calculated for C_18_H_20_ClN_7_S, 402.1268 [M+H]^+^; found, 402.1265.

##### 4-(((3-(2-(Pyridin-2-ylamino)ethyl)-5-thioxo-1,5-dihydro-4*H*-1,2,4-triazol-4-yl)imino)methyl)benzoic acid (**13**)

Prepared from carboxybenzaldehyde. Yield 65% (0.35 g), yellowish-brown crystals. m.p. 279–280 °C. IR (KBr) ν_max_ (cm^−1^): 1277 (C=S); 3123, 3264 (NH). ^1^H NMR (400 MHz, DMSO-*d*_6_): *δ* = 3.00 (t; 2H; *J* = 6.8 Hz; H_8_), 3.79 (q; 2H; *J* = 6.8 Hz; *J* = 6.8 Hz; H_7_), 6.86 (t; H; *J* = 8.2 Hz; NH), 7.10 (d; H; *J* = 8.2 Hz; H_6_), 7.21–7.47 (m, H, H_5_), 7.74 (d, H, H_3_), 7.87–7.90 (m, 2H, H_4,13_), 8.02–8.10 (m, 3H, H_14,16,17_), 8.18 (s, H, H_11_), 10.57 (s, H, OH), 12.00 (s, H, NH). ^13^C NMR (101 MHz, DMSO-*d*_6_): *δ* = 24.0 (C_8_), 38.4 (C_7_), 112.2, 127.6, 127.7, 129.6, 131.5, 138.2, 141.8, 143.5, 147.9, 149.6, 152.6 (C_1,3,4,5,6,9,11,13,14,15,16,17_), 166.0 (C_10_), 167.0 (C_18_). HRMS (ESI+): *m*/*z* calculated for C_17_H_16_N_6_O_2_S, 369.1134 [M+H]^+^; found, 369.1386.

##### 4-(((3-(2-((5-Chloropyridin-2-yl)amino)ethyl)-5-thioxo-1,5-dihydro-4*H*-1,2,4-triazol-4-yl)imino)methyl)benzoic acid (**14**)

Prepared from carboxybenzaldehyde. Yield 53% (0.42 g), light yellow crystals. m.p. 241–242 °C. IR (KBr) ν_max_ (cm^−1^): 1276 (C=S), 2940; 3063 (NH). ^1^H NMR (400 MHz, DMSO-*d*_6_): *δ* = 3.03 (t; 2H; *J* = 6.8 Hz; H_8_), 3.61 (t; 2H; *J* = 6.8 Hz; H_7_), 6.46 (d; H; *J* = 6.8 Hz; H_6_), 7.08 (s, H, NH), 7.32 (d; H; *J* = 6.8 Hz; H_5_), 7.85–7.91 (m, 3H, H_3,13,17_), 7.97–8.14 (m, 3H, H_11,14,16_), 10.14 (s, H, OH), 13.86 (s, H, NH). ^13^C NMR (101 MHz, DMSO-*d*_6_): *δ* = 24.7 (C_8_), 38.3 (C_7_), 110.5, 117.6, 128.5, 129.6, 129.9, 133.9, 136.0, 137.2, 143.9, 150.0, 156.3 (C_1,3,4,5,6,9,11,13,14,15,16,17_), 161.2 (C_10_), 166.7 (C_18_). HRMS (ESI+): *m*/*z* calculated for C_17_H_15_ClN_6_O_2_S 403.0745 [M+H]^+^, found 403.0741.

##### 5-(2-(Pyridin-2-ylamino)ethyl)-4-((pyridin-3-ylmethylene)amino)-2,4-dihydro-3*H*-1,2,4-triazole-3-thione (**15**)

Prepared from 3-pyridinecarbaldehyde. Yield 61% (0.29 g), bright yellow crystals. m.p. 191–192 °C. IR (KBr) ν_max_ (cm^−1^): 1225 (C=S); 3133; 3266 (NH). ^1^H NMR (400 MHz, DMSO-*d*_6_): *δ* = 2.76–2.97 (m, 2H, H_8_), 3.71–3.76 (m, 2H, H_7_), 5.52 (s, H, NH), 6.38–6.46 (m, H, H_4_), 6.52–6.63 (m, H, H_6_), 7.28–7.34 (m, H, H_5_), 7.95 (s, H, H_3_), 8.39–8.42 (m, 2H, H_16,17_), 8.70 (s, 2H, H_13,15_), 9.09 (s, H, H_11_), 11.93 (s, H, NH). ^13^C NMR (101 MHz, DMSO-*d*_6_): *δ* = 25.1 (C_8_), 30.6 (C_7_), 104.9, 111.7, 118.3, 123.9, 130.1, 133.9, 136.6, 141.4, 147.5, 148.8, 150.6, 156.2 (C_1,3,4,5,6,9,11,12,13,15,16,17_), 175.4 (C_10_). HRMS (ESI+): *m*/*z* calculated for C_15_H_15_N_7_S, 326.1189 [M+H]^+^; found, 326.1183.

##### 5-(2-((5-Chloropyridin-2-yl)amino)ethyl)-4-((pyridin-3-ylmethylene)amino)-2,4-dihydro-3*H*-1,2,4-triazole-3-thione (**16**)

Prepared from 3-Pyridinecarbaldehyde. Yield 71% (0.51g), greyish crystals. m.p. 160–161 °C. IR (KBr) ν_max_ (cm^−1^): 1280 (C=S), 2930, 3130 (NH). ^1^H NMR (400 MHz, DMSO-*d*_6_): *δ* = 3.26 (t; 2H; *J* = 6.8 Hz; H_8_), 3.80 (t; 2H; *J* = 6.8 Hz; H_7_), 6.99 (d; H; *J* = 6.8 Hz; H_6_), 7.12 (s, H, NH), 7.24 (d; H; *J* = 6.8 Hz; H_5_), 7.80–7.83 (m, 2H, H_15,16_), 7.86–8.07 (m, 3H, H_3,13,17_), 8.81 (s, H, H_11_), 13.95 (s, H, NH). ^13^C NMR (101 MHz, DMSO-*d*_6_): *δ* = 24.1 (C_8_), 30.7 (C_7_), 111.5, 114.3, 117.2, 120.0, 127.9, 130.0, 134.6, 143.5, 152.9 (C_1,3,4,5,6,9,11,12,13,15,16,17_), 165.8 (C_10_). HRMS (ESI+): *m*/*z* calculated for C_15_H_14_ClN_7_S, 360.8439 [M+H]^+^; found, 360.0795.

##### 4-(((1-Methyl-1*H*-pyrazol-3-yl)methylene)amino)-5-(2-(pyridin-2-ylamino)ethyl)-2,4-dihydro-3*H*-1,2,4-triazole-3-thione (**17**)

Prepared from 1-Methyl-1*H*-pyrazole-4-carbaldehyde. Yield 53% (0.26 g), light brown crystals. m.p. 197–198 °C. IR (KBr) ν_max_ (cm^−1^): 1280 (C=S); 3105; 3315 (NH). ^1^H NMR (400 MHz, DMSO-*d*_6_): *δ* = 1.91 (s, 3H, H_15_), 2.95 (t; 2H; *J* = 6.4 Hz; H_8_), 3.61 (q; 2H; *J* = 12.8 Hz; H_7_), 6.40 (t; H; *J* = 6.4 Hz; H_4_), 6.42–6.45 (m, H, H_6_), 6.64 (t; H; *J* = 6.4 Hz; NH), 6.80 (s, H, H_13_), 7.29 (t, H, *J* = 7.2 Hz, H_5_), 7.86 (s, H, H_14_), 7.92 (s, H, H_3_), 9.92 (s, H, H_11_), 11.97 (s, H, NH). ^13^C NMR (101 MHz, DMSO-*d*_6_): *δ* = 21.1 (C_15_), 25.3 (C_8_), 37.5 (C_7_), 104.8, 108.3, 111.7, 133.2, 136.6, 145.5, 147.4, 149.7, 157.3, 158.4 (C_1,3,4,5,6,9,11,12,13,14_), 161.1 (C_10_). HRMS (ESI+): *m*/*z* calculated for C_14_H_16_N_8_S, 329.1298 [M+H]^+^; found, 329.1296.

##### 5-(2-((5-Chloropyridin-2-yl)amino)ethyl)-4-(((1-methyl-1*H*-pyrazol-3-yl)methylene)amino)-2,4-dihydro-3*H*-1,2,4-triazole-3-thione (**18**)

Prepared from 1-methyl-1*H*-pyrazole-4-carbaldehyde. Yield 79% (0.57 g), brownish crystals. m.p. 157–158 °C. IR (KBr) ν_max_ (cm^−1^): 1278 (C=S), 2941; 3114 (NH). ^1^H NMR (400 MHz, DMSO-*d*_6_): *δ* = 2.94 (t; 2H; *J* = 6.4 Hz; H_8_), 2.54 (s, 2H, H_7_), 3.93 (t; 3H; *J* = 6.4 Hz; H_15_), 6.39 (d; H; *J* = 8.8 Hz; H_6_), 6.74 (s, H, H_13_NH), 6.87 (t; H; *J* = 6.4 Hz; NH), 7.32 (d; H; *J* = 8.8 Hz; H_5_), 7.82 (s, H, H_3_), 7.86 (s, H, H_14_), 9.81 (s, H, H_11_), 13.71 (s, H, NH). ^13^C NMR (101 MHz, DMSO-*d*_6_): *δ* = 21.3 (C_15_), 25.2 (C_8_), 38.1 (C_7_), 105.1, 110.0, 117.72, 133.3, 136.7, 145.4, 145.6, 150.0, 157.2, 157.8 (C_1,3,4,5,6,9,11,12,13,14_), 161.3 (C_10_). HRMS (ESI+): *m*/*z* calculated for C_14_H_15_ClN_8_S, 363.0908 [M+H]^+^; found, 363.1249.

##### 4-((2-Hydroxybenzylidene)amino)-5-(2-(pyridin-2-ylamino)ethyl)-2,4-dihydro-3*H*-1,2,4-triazole-3-thione (**19**)

Prepared from 2-hydroxybenzaldehyde. Yield 54% (0.27 g), light yellow crystals. m.p. 177–178 °C. IR (KBr) ν_max_ (cm^−1^): 1261 (C=S); 3131; 3245 (NH). ^1^H NMR (400 MHz, DMSO-*d*_6_): *δ* = 2.98 (t; 2H; *J* = 6.4 Hz; H_8_), 3.60 (q; 2H; *J* = 6.4 Hz; *J* = 12.4 Hz; H_7_), 6.38–6.44 (m, 2H, H_4,6_), 6.65 (t; H; *J* = 8.00 Hz; NH), 6.89–7.01 (m, 3H, H_14,16,17_), 7.28 (t; H; *J* = 7.6 Hz; H_5_), 7.41 (t; H; *J* = 7.6 Hz; H_15_), 7.86 (d; H; *J* = 7.6 Hz; H_3_), 7.92 (s, H, H_11_), 10.16 (s, H, OH), 11.64 (s, H, NH). ^13^C NMR (101 MHz, DMSO-*d*_6_): *δ* = 25.3 (C_8_), 37.6 (C_7_), 108.3, 111.6, 116.6, 117.3, 118.5, 119.2, 119.6, 122.3, 127.2, 129.2, 134.1, 136.4, 136.5, 147.4, 149.8, 158.4, 160.3 (C_1,3,4,5,6,9,11,12,13,14,15,16,17_), 161.2 (C_10_). HRMS (ESI+): *m*/*z* calculated for C_16_H_16_N_6_OS, 341.1185 [M+H]^+^; found, 341.1183.

##### 5-(2-((5-Chloropyridin-2-yl)amino)ethyl)-4-((2-hydroxybenzylidene)amino)-2,4-dihydro-3*H*-1,2,4-triazole-3-thione (**20**)

Prepared from 2-hydroxybenzaldehyde. Yield 73% (0.55 g), yellowish brown crystals. m.p. 158–159 °C. IR (KBr) ν_max_ (cm^−1^): 1266 (C=S), 2930, 3054 (NH). ^1^H NMR (400 MHz, DMSO-*d*_6_): *δ* = 2.97 (t; 2H; *J* = 6.8 Hz; H_8_), 3.58 (t; 2H; *J* = 6.8 Hz; H_7_), 6.42 (d; H; *J* = 6.8 Hz; H_6_), 6.89 (t; H; *J* = 6.8 Hz; NH), 6.93–7.00 (m, 3H, H_14_,_16,17_), 7.30 (d; H; *J* = 6.8 Hz; H_5_), 7.39 (t; H; *J* = 7.6 Hz; H_15_), 7.76–7.80 (m, H, H_3_), 7.90, 7.96 (2s, H, H_11_), 10.14, 10.27 (2s, H, OH), 11.88 (s, H, NH). ^13^C NMR (101 MHz, DMSO-*d*_6_): *δ* = 25.1 (C_8_), 37.9 (C_7_), 109.8, 116.7, 117.4, 118.5, 119.2, 122.4, 127.0, 128.9, 134.0, 136.4, 145.3, 149.6, 157.0, 158.8 (C_1,3,4,5,6,9,11,12,13,14,15,16,17_), 160.4 (C_10_). Analysis calculated for C_16_H_16_N_6_OS: C, 51.27; H, 4.03; N, 22.42. Found: C, 51.16; H, 3.99; N, 22.01.

##### 5-(2-((5-Chloropyridin-2-yl)amino)ethyl)-4-((4-(methylthio)benzylidene)amino)-2,4-dihydro-3*H*-1,2,4-triazole-3-thione (**21**)

Prepared from 4-(methylthio)benzaldehyde. Yield 58% (0.47 g), reddish crystals. m.p. 152–153 °C. IR (KBr) ν_max_ (cm^−1^): 1282 (C=S), 2917; 3130 (NH). ^1^H NMR (400 MHz, DMSO-*d*_6_): *δ* = 2.45 (s, 3H, H_18_), 2.53 (t; 2H; *J* = 8.8 Hz; H_8_), 2.88 (t; 2H; *J* = 8.8 Hz; H_7_), 6.49 (d; H; *J* = 8.8 Hz; H_6_), 6.88 (t; H; *J* = 8.8 Hz; NH), 7.23–7.25 (m, 2H, H_14,16_), 7.70 (d; H; *J* = 8.8 Hz; H_5_)_,_ 7.79–7.87 (m, 3H, H_3,13,17_), 9.78, 9.89 (2s, H, H_11_), 11.51, 11.82 (2s, H, NH). ^13^C NMR (101 MHz, DMSO-*d*_6_): *δ* = 14.1 (C_18_), 25.0 (C_8_), 37.71 (C_8_), 110.0, 117.7, 125.3, 129.0, 130.1, 132.8, 134.9, 136.7, 138.5, 145.5, 147.6, 150.8, 157.3, 166.0 (C_1,3,4,5,6,9,11,12,13,14,15,16,17_), 174.58 (C_10_). HRMS (ESI+): *m*/*z* calculated for C_17_H_17_ ClN_6_S_2_, 405.0724 [M+H]^+^; found, 405.0722.

### 2.2. Biology

#### 2.2.1. Determination of Antimicrobial Activity

The following test microorganisms were used for the investigation of the antibacterial and antifungal properties of the synthesized compounds: the bacteria *Escherichia coli* B-906, *Staphylococcus aureus* 209-P, and *Mycobacterium luteum* B-917 and the fungi *Candida tenuis* VKM Y-70 and *Aspergillus niger* F-1119. These test cultures belong to different systematic groups of microorganisms. *E. coli* is an opportunistic Gram-negative bacterium. *S. aureus* is a representative of opportunistic Gram-positive bacteria. *M. luteum* is a phytopathogenic Gram-positive bacterium. *C. tenuis* is a representative of yeast fungi, and *A. niger* represents mold fungi.

The diffusion method [27] and the serial dilution method [28] were used for the determination of antimicrobial activity. Vancomycin (an antibacterial substance) and nystatin (an antifungal substance) were used as the reference agents (controls) in the study. Each experiment was carried out in triplicate. The results of the antimicrobial evaluation are presented in Table 1 and Table 2.

##### Determination of Antimicrobial Activity by the Diffusion Assay

The antibacterial and antifungal activity of the synthesized compounds was evaluated by diffusion in peptone on a nutrient medium (meat-extract agar for bacteria, wort agar for fungi). A total of 20 millilitres of the nutrient medium was added to Petri plates for all test cultures. The microbial loading was 10^9^ cells (spores)/1 mL. Whatmann filter disks with 0.1 and 0.5% of the test compounds were placed on the Petri plates. The incubation period for the bacterial cultures was 24 h at 35 °C, while the fungal cultures were incubated for 48–72 h at 28–30 °C. The antimicrobial effect of the compounds tested was estimated by measuring the zone diameter (mm) of the microorganism growth inhibition (Table 1).

##### Determination of Minimal Inhibitory Concentrations (MICs) by the Serial Dilution Assay

The test compound was dissolved in the solvent (DMSO) at the necessary concentration. A flat-bottomed 96-well tissue culture plate was used for testing. A certain volume of the compound solution was introduced to the nutrient medium (meat-extract agar for bacteria and wort for fungi). The inoculum of bacteria and fungi was inoculated in the nutrient medium. The incubation was carried out at 37 °C for the bacteria and at 30 °C for the fungi. The duration of incubation was 24–72 h. The results were estimated based on the presence or absence of microorganisms’ growth in the flat-bottomed 96-well tissue culture plate and are presented as minimal inhibitory concentrations (MICs) (Table 2).

#### 2.2.2. Determination of Anticancer Activity

##### Human Cell Cultures

MDA-MB-231 (breast/mammary gland; adenocarcinoma) and U-87 (brain; derived from malignant gliomas, likely glioblastoma) cells were obtained from ATCC. The cells were cultured in Dulbecco’s modified Eagle’s medium (Gibco, Paisley, UK) containing 10% fetal bovine serum (Gibco, Paisley, UK) and 1% penicillin (10,000 U/mL)–streptomycin (10 μg/mL) (Gibco, Grand Island, NY, USA) at 37 °C in humidified 5% CO_2_ conditions. The cells were cultivated for no more than 30 passages and routinely tested for mycoplasma.

##### Cell Viability Assay

MDA-MB-231 and U-87 cells were seeded at 5 × 10^3^ cells per well in 96-well plates. The cells were treated with a 100 μM solution of the tested compounds dissolved in DMSO. The same volume of DMSO as in the tested samples was used as a negative control. Each test for each compound was repeated in triplicate wells and incubated at 37 °C for 72 h. Cell viability was measured using the MTT (3-(4,5-dimethylthiazol-2-yl)-2,5-diphenyltetrazolium bromide) method. This colorimetric analysis is based on the reduction of yellow tetrazolium salt to pink formazan crystals by metabolically active cells. Briefly, 0.4 mg/mL of MTT solution was added to each well and the cells were incubated at 37 °C for 4 h. After incubation, the MTT solution was removed and replaced with 100 μL of DMSO to dissolve the formazan crystals. The absorbance was measured at 570 nm using a microplate-reading spectrophotometer (Sunrise, TECAN), and the effect of the compounds on the cell viability was calculated as follows: the percentage of cell viability = (A_treatment_ − A_blank_)/(A_control_ − A_blank_) × 100%, where A_experimental_ is the absorbance of the cells treated with the tested compounds; A_blank_ is the blank sample absorbance (only culture medium; positive control); and A_control_ is the absorbance of the cells treated with DMSO (negative control). The IC_50_ value determination of the selected compounds was performed using the same MTT cell viability assay. Serial dilutions of each compound from 100 µM to 10 µM were made in medium and added to the cells in triplicate. The MTT assay was repeated three times. The obtained results were calculated using GraphPad Prism 8 software.

### 2.3. Molecular Docking

Molecular docking for compounds **15**, **16**, and **21** was carried out using the software package *SchrödingerSuite 2024-1* [29]. The structures of the target proteins EGFR (PDB: 4HJO), VEGFR (PDB: 4AGD, 4ASD, 3EWH), HER2 (PDB: 3RCD), BRAF (PDB: 1UWH, 5VAM, 4G9C), MEK (PDB: 4U7Z), SCR (PDB: 3F3V), and CDK5 (PDB: 1UNL) were retrieved from the Protein Data Bank (PDB) [30]. The structures of the kinase drugs Dasatinib, Sorafenib, and Vemurafenib were retrieved from respective PDBs, where they were identified as binding to the respective kinases. The ligands were prepared using the LigPrep software version 3.9 [31]. The preparation of the target protein was carried out using Protein Preparation Wizard [32]. The generation of the ligand–receptor bonding area was carried out using the Grid Generation of the Glide Maestro module [33].

## 3. Results and Discussion

### 3.1. Chemistry

**Scheme 1:**
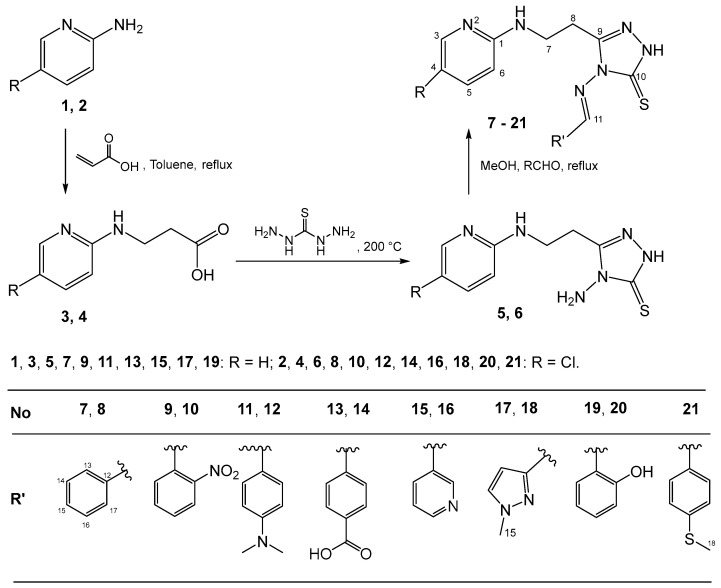
Synthesis of hydrazones **7**–**21**.

The target hydrazones **7**–**21** containing a pyridine ring were synthesized as shown in Scheme 1. The reaction of 2-aminopyridine **1** and **2** with acrylic acid in toluene under the reflux conditions gave the corresponding β-alanines **3** and **4** [26]. Compounds **3** and **4** were melted at 200 °C with thioacetohydrazide to provide corresponding 4-amino-1,2,4-triazole-3-thiones **5** and **6** in 74–76% yield. Target 4-substituted-5-(2-(pyridine-2-ylamino)ethyl)-2,4-dihydro-3*H*-1,2,4-triazole-3-thiones **7**, **9**, **11**, **13**, **15**, **17**, and **19**, and their chloro analogs **8**, **10**, **12**, **14**, **16**, **18**, **20**, and **21** were synthesized by the reaction of **5** and **6** with corresponding aldehydes in methanol in the presence of a catalytic amount of hydrochloric acid under reflux conditions in a 53–85% yield.

The structures of the synthesized compounds were confirmed by ^1^H and ^13^C NMR, as well as HRMS data (Supplementary Materials). In the ^1^H NMR spectra of **5** and **6**, the triplet ascribed to the protons of the methylene group adjacent to the C=O group was 2.89 and 2.87 ppm, respectively, and a quadruplet at 3.59 ppm was attributed to the protons of the methylene group adjacent to the NH group. A singlet at ~5.5 ppm, integrated for two protons, confirmed the presence of the amino group. The presence of the NH group in the triazole ring was proved by the singlet at ~13.45 ppm. In the ^13^C NMR spectra of **5** and **6**, a carbon resonance at ~165.8 ppm and 165.9 ppm confirmed the presence of the C=S group in the 1,2,4-triazole-3-thione moiety. In the ^1^H NMR spectra of the target hydrazones **7**–**21**, the NH_2_ singlet was replaced by the proton singlet of the CH group in the range of 7.92–10.87 ppm. The incorporation of the moieties from the respective aldehydes were confirmed by the corresponding proton resonances in the ^1^H NMR spectra of **7**–**21**.

### 3.2. Biology

#### 3.2.1. Antimicrobial Activity

The synthesized compounds **5**–**21** were evaluated for their antimicrobial properties against the bacterial strains *E. coli*, *S. aureus*, and *M. luteum* as well as the fungi *C. tenuis* and *A. niger* by the agar diffusion method and serial dilutions method. The antimicrobial properties of the synthesized compounds were compared with the action of vancomycin (an antibacterial drug) and nystatin (an antifungal drug) as reference drugs.

As seen from the results of the agar diffusion test (Table 1), the strains of *E. coli* and *S. aureus* bacteria were insensitive to the action of the majority of the synthesized compounds. Compound **16**, which had two pyridine rings in its structure, was the only very active exception, and the diameter of its growth inhibition zone (14.4 mm) at a concentration of 0.5% was close to the effect of the vancomycin. The antibacterial effect (18 mm) of 4-amino-1,2,4-triazole-3-thione **6**, bearing a 5-chloropyridine moiety against the test culture of *M. luteum*, was at the same level as that of the reference drug vancomycin (18 mm). It is interesting to note that analog **5**, bearing a chloro substituent, was less active against the *M. luteum* strain than compound **6** at a concentration of 0.5%. The sensitivity of the *M. luteum* strain to the action of compounds **7**, **12**, **21**, **10**, **16**, and **18** varied from moderate to low at a concentration of 0.5%. The agar diffusion assay revealed that only a few compounds exhibited a medium influence on *C. tenuis* and *A. niger* among the test fungi cultures. In particular, compound **6** and the dimethylaminobenzylidene moiety-bearing 5-chloropyridine derivative **12**, at a concentration of 0.5%, inhibited the growth of the *C. tenuis* strain with the diameter of the growth inhibition zone being 15.0–15.4 mm. The test cultures of *C. tenuis* and *A. niger* were insensitive to the action of compounds **19** and **20** bearing the 2-hydroxybenzylidene moiety at concentrations of 0.5% and 0.1%. The 5-Chloropyridine derivative **18**, bearing the 1-methylpyrazole moiety, inhibited the growth of the *A. niger* strain the most among the tested compounds at a concentration of 0.5% (12 mm).

The serial dilution method was used to determine the exact minimum inhibitory concentrations (MICs) of the synthesized compounds **5**–**21** (Table 2). The Gram-negative bacterium *E. coli* was sensitive to the action of compounds **6** and **20** at MICs of 125.0 and 500.0 μg/mL, respectively. The Gram-positive bacterium *S. aureus* demonstrated sensitivity to compounds **6** and **16** at an MIC of 250.0 μg/mL. The test culture of *M. luteum* was sensitive to both 4-amino-1,2,4-triazole-3-thiones **5** and **6**, as well as the benzene ring-bearing hydrazone **7**, at an MIC = 7.8 μg/mL, which was the same level of activity as that of the vancomycin. It was observed that compounds **8**, **10**, **16**, and **21** exhibited activity against *M. luteum* at an MIC of 3.9 µg/mL. The MICs for the other screened compounds were found to be 15.6–250.0 µg/mL.

Among the tested fungal cultures, the strain *C. tenuis* exhibited high sensitivity to the action of compounds **6** and **7** at an MIC = 0.9 µg/mL; **5**, **8**, and **16** at an MIC = 1.9 µg/mL; **10** and **21** at an MIC = 3.9 µg/mL; and **18** at an MIC = 7.8 µg/mL. It should be noted that the inhibitory antifungal effect of all these compounds was more profound than that of the reference drug, nystatin. From the point of view of the structure–activity relationship, no consistent pattern was observed regarding the presence or absence of the chlorine atom in the pyridine ring moiety. Between 4-amino-1,2,4-triazole-3-thiones **5** and **6**, compound **6**, bearing an unsubstituted pyridine ring, was more active than the 5-chloropyridine derivative **5**, while hydrazone **7**, bearing a 5-chloropyridine moiety, was more active than its unsubstituted analog **8**. The 5-chloropyridine derivative **12**, bearing the dimethylaminobenzylidene moiety, demonstrated an inhibitory effect on the level of nystatin at an MIC = 15.6 µg/mL, while analog **11**, bearing no chlorine atom, was not active at all. The MIC values of other compounds were in the range of 31.2–500 µg/mL. The MIC values of the synthesized compounds against *A. niger* were found to be within the range of 31.2–500 µg/mL.

In summary, the structure–activity relationship analysis revealed that the conversion of the hydrazide fragment into the hydrazone moiety preserved the antibacterial properties against the test culture *M. luteum* and the strain *C. tenuis*. The introduction of a phenyl substituent into the ylidene moiety increased the antibacterial activity of compound **8** against the test culture of *M. luteum* and the antifungal effect against *C. tenuis*. Replacing the phenyl fragment with the pyridine **16** or 4-methylthiophenyl moiety **21** also increased the antibacterial activity against the mentioned bacteria compared to the starting compounds **5** and **6**.

It can be concluded that 4-amino-1,2,4-triazole-3-thiones **5** and **6** and hydrazones **7**, **8**, **10**, **12**, **16**, and **21** have been identified as the compounds with the strongest antimicrobial properties among the compounds tested. These compounds have been demonstrated to possess high antibacterial activity against the test culture *M. luteum* and antifungal activity against the strain *C. tenuis*. Therefore, they could be considered for further in-depth studies as new potential antimicrobial agents.

#### 3.2.2. Evaluation of Cancer Cell Viability

Two different human cancer cell lines were used for this study, namely, triple-negative breast cancer MDA-MB-231 and glioblastoma U-87. Triple-negative breast cancer and glioblastoma are known as aggressive cancers with high resistance to many available chemotherapeutic drugs. The patients of both cancer types have a poor survival rate, and their treatment has some limitations [34,35]. Therefore, the elucidation of innovative and effective drugs can help to improve existing or even offer new treatment solutions.

Recent studies have shown that 1,2,4-triazole-based compounds have great potential for cancer treatment due to their important chemical and biological properties [36,37]. Anticancer agents have different mechanisms of action, such as alkylating agents, biological response modifiers, antiandrogens, topoisomerase inhibitors or protein kinase inhibitors, etc. [38]. It is known that protein kinases involved in cell signaling pathways are dysregulated in cancer cells. Therefore, kinases have recently become one of the most important targets for anticancer drugs [39,40]. Some studies have revealed that compounds containing the 1,2,4-triazole moiety can act as kinase inhibitors and may be promising for cancer treatment. For example, the 5-mercapto-1,2,4-triazole derivative inhibits phosphoinositide PI3K kinases on human melanoma A375, breast carcinoma MDA-MB-231, lung carcinoma A549, and murine melanoma B164A5 cell lines [41]; 1,2,4-triazole-3,5-diamine analogs have been synthesized as cyclin-dependent kinase CDK1 and CDK2 inhibitors [42].

**Figure 1:**
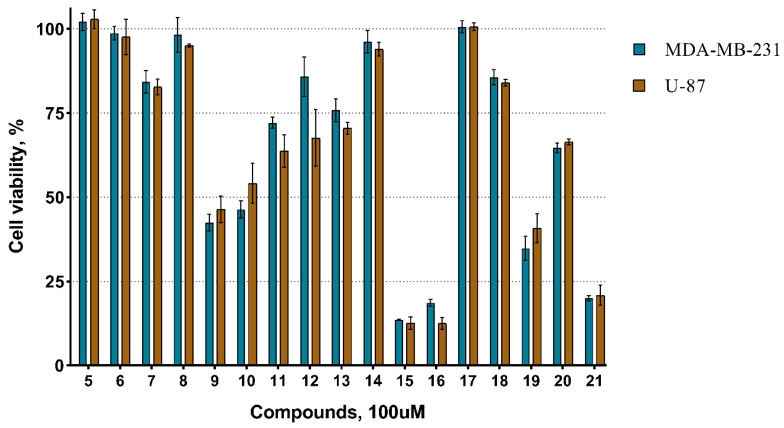
The effect of compounds **5**–**21** on the viability of the MDA-MB-231 and U87 cells. The data represent the means ± SD from three independent experiments (*n* = 3).

The effect of newly synthesized compounds on cell viability is the initial stage of a typical anticancer drug discovery process. The effect of the synthesized 1,2,4-triazole-3-thione derivatives **5**–**21** on cell viability was screened using triple-negative breast cancer MDA-MB-231 and glioblastoma U-87 cell lines by MTT assay at 100 µM, and the concentration was selected based on studies described earlier [20]. The obtained results showed that each of the tested compounds had a different cytotoxic effect on the MDA-MB-231 and U-87 cells (Figure 1). Compounds **5**–**8**, **14**, **17**, and **18** showed relatively low effects (0–20%) on the viability of both the MDA-MB-231 and U-87 cell lines. This was not surprising, because the used cell lines MDA-MB-231 and U-87 were derived from human triple-negative breast cancer and glioblastoma, respectively. Both types of these tumors are known to be the most aggressive cancers with high resistance to many available chemotherapy drugs, resulting in the poor survival of patients [34,35]. Cell treatments with compounds **10**–**13** and **20** reduced the viability by 20–50%, indicating their moderate cytotoxic effects on the breast and glioblastoma cells.

The treatment of cells with solutions of compounds **9**, **15**, **16**, **19**, and **21** reduced cell viabilities by more than 50%. Hydrazone **9**, bearing a 2-nitrobenzylidene moiety, and hydrazone **19**, bearing a 2-hydroxybenzylidene moiety, had similar cytotoxic effects on both cell types. The number of viable cells after incubation with compounds **9** and **19** in the breast cancer cells was up to 42.5 ± 2.46% and 34.8 ± 3.51%, respectively. In the case of the U-87 cells, compounds **9** and **19** reduced the cytotoxic sensitivity of the glioblastoma cells to 46.3 ± 3.97%, and 40.8 ± 4.28%, respectively.

Hydrazones **15** and **16**, bearing two pyridine rings in their molecules, and the 5-chloropyridine derivative **21**, bearing a 4-methylthiobenzylidene moiety, were identified as the most effective compounds in reducing cell viability. They reduced the viability of the MDA-MB-231 cells to 13.6 ± 0.18%, 18.5 ± 1.04%, and 19.9 ± 0.82% and that of U-87 cells to 12.5 ± 1.81%, 12.5 ± 1.73%, and 20.8 ± 2.98%, respectively. The activity of the 4-methylthiobenzylidene derivative **21** was similar on both the breast (19.9 ± 0.82%) and glioblastoma (20.8 ± 2.98%) cells. The most promising of them was the compound **15**; the treatment with this solution resulted in 13.6 ± 0.18% and 12.5 ± 1.81% reductions in the viable breast and glioblastoma cells, respectively. It is interesting to note that the 5-chloropyridine derivative **16** was less active than its unsubstituted analog **15**.

**Figure 2:**
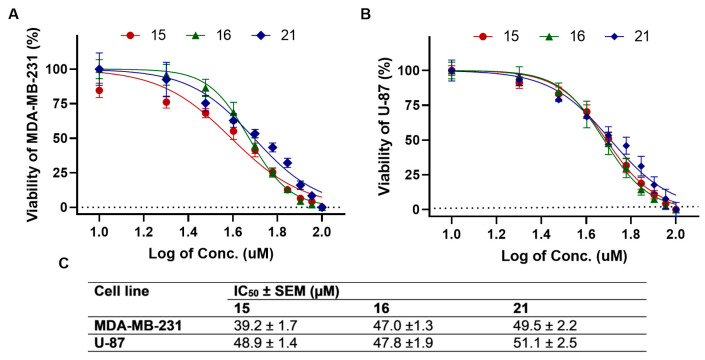
Summary of determination of half-maximal inhibitory concentrations (IC_50_) for compounds **15**, **16**, and **21**. Dose–response curves (averages ± SEMs, *n* = 3) for MDA-MB-231 (**A**) and U-87 (**B**) cell lines treated with compounds **15**, **16**, and **21**. (**C**) IC_50_ value of compounds in tested cells.

The most active compounds, **15**, **16**, and **21**, were selected for the further study of their efficacy by determining their half-maximal inhibitory concentrations (IC_50_) that reduce the cell viability by 50% (Figure 2). To estimate the IC_50_ values, both the MDA-MB-231 and U-87 cell lines were treated with solutions of compounds **15**, **16**, and **21**, and the same MTT assay was used. The IC_50_ values of the selected compounds are presented in Figure 2C. The lowest IC_50_ value (39.2 ± 1.7 μM) was achieved by compound **15** against the MDA-MD-231 cells. Furthermore, this compound showed slightly higher selectivity against the MDA-MB-231 cells compared to the U-87 cells (IC_50_ values = 39.2 ± 1.7 μM against MDA-MB-231 and 48.9 ± 1.4 μM against U-87 cells). Dose–response analysis (Figure 2A,B) revealed that compounds **16** and **21** possessed a similar cytotoxicity and were non-selective in the breast cancer and glioblastoma cells.

In summary, hydrazones **15**, **16**, and **21** were identified as possessing significant cytotoxicity in the tested breast and glioblastoma cells. The most promising candidate among them, hydrazone **15**, bearing two unsubstituted pyridine rings, demonstrated exceptional cytotoxicity and selectivity against the MDA-MB-231 which was just slightly higher than that against the U-87 cells. However, the reported results represent an initial step in the anticancer drug discovery process. Different compounds in the tested cell lines obtained from different tumors might affect different cellular pathways; therefore, which cellular pathways are affected by these compounds remains to be determined in more detailed future studies.

### 3.3. In Silico Evaluation of Potential Compound Action Targets

Molecular docking was conducted for compounds **15**, **16**, and **21**, which exhibited significant experimental cytotoxicity against the *MDA-MB-231* and *U-87* cancer cell lines at the next stage. This was conducted to estimate plausible anticancer mechanisms.

In the treatment of breast cancer and glioblastoma, kinase inhibitors have been shown to be effective therapeutics [43,44]. Tyrosine kinase inhibitors targeting receptors (for example, EGFR, HER2, and VEGFR inhibitors) [45], non-receptor tyrosine kinases (like SRC and ACK-1 inhibitors) [46,47], and serine–threonine protein kinase inhibitors (such as BRAF and MEK inhibitors) [48] have a significant impact on the progression of breast cancer and glioblastoma. Therefore, the modeling of the interaction between compounds **15**, **16**, and **21** and the active centers of selected receptor (EGFR-PDB: 4HJO; VEGFR-PDB: 4AGD, 4ASD, 3EWH; HER2-PDB: 3RCD; CDK5-PDB: 1UNL) and non-receptor (SCR-PDB: 3F3V) tyrosine kinases, as well as serine–threonine (BRAF-PDB: 1UWH, 5VAM, 4G9C; MEK-PDB: 4U7Z) protein kinases, was carried out. The multi-target kinase inhibitor drugs dasatinib, sorafenib, and vemurafenib were selected as standard ligands for binding at the sites of the proteins selected for the treatment of the breast cancer and glioblastoma. The results of the docking procedure indicated a high degree of binding to the four active centers of the selected serine–threonine protein kinases (Table 3).

**Figure 3:**
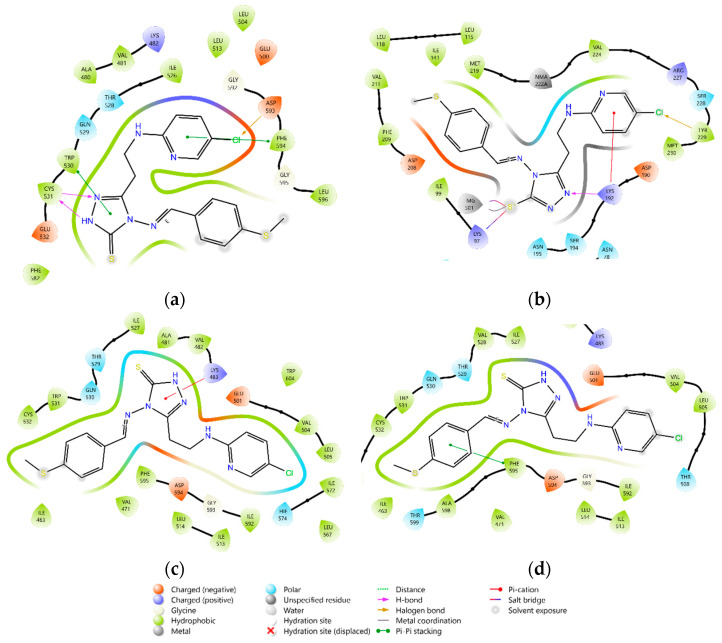
The 2D interaction of compound **21** with amino acid residues in the active sites of corresponding proteins: (**a**)—**21** with BRAF (PDB: 1UWH); (**b**)—**21** with MEK (4U7Z); (**c**)—**21** with BRAF (PDB: 5VAM); and (**d**)—**21** with BRAF (PDB: 4G9C).

**Figure 4:**
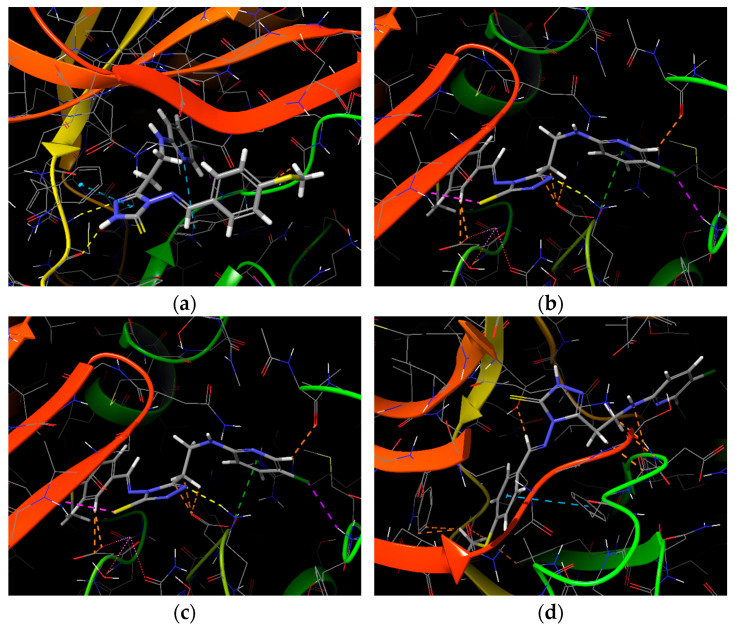
The 3D interaction of compound **21** with amino acid residues in the active sites of corresponding proteins: (**a**)—**21** with BRAF (PDB: 1UWH); (**b**)—**21** with MEK (4U7Z); (**c**)—**21** with BRAF (PDB: 5VAM); and (**d**)—**21** with BRAF (PDB: 4G9C).

As shown in Table 3, the compounds **15**, **16**, and **21** showed a high affinity for four types of protein kinase sites, *1UWH, 5VAM, 4G9C* (BRAF), and *4U7Z* (MEK), with binding scores ranging from −9.103 to −10.122 kcal/mol. The glide emodel values of these compounds were comparable to or exceed the values of the standard ligands. The 4-methylthiobenzylidene derivative **21** was identified as possessing the highest affinity among the compounds under investigation. The 2D and 3D visualizations of the binding at the protein sites are presented in Figure 3 and Figure 4.

Therefore, the results of the molecular modeling suggest that compounds **15**, **16** and **21** were probably acting on the mitogen-activated protein kinase pathway through the inhibition of the BRAF and MEK serine–threonine protein kinases. This could be the key to in-depth studies for the discovery of new effective anticancer drugs for the treatment of breast cancer and glioblastoma.

## 4. Conclusions

4-Amino-1,2,4-triazole-3-thiones **5** and **6** and a series of target 4-substituted-5-(2-(pyridine-2-ylamino)ethyl)-2,4-dihydro-3*H*-1,2,4-triazole-3-thiones and their chloro analogs **7**–**21** were synthesized, and their antimicrobial and anticancer properties were evaluated using in vitro and in silico methods. The screening of the antibacterial activity of the synthesized compounds revealed that hydrazones **8**, **10**, **16**, and **21** possessed antibacterial activity (MIC 3.9 µg/mL) against the strain *M. luteum* and were stronger antibacterial agents than the reference drug vancomycin (MIC 7.8 µg/mL). The evaluation of the antifungal activity of the synthesized compounds against *C. tenuis* and *A. niger* indicated that compounds **6** and **7** were exceptionally active against *C. tenuis* (MIC 0.9 µg/mL). The cell viability screening of the synthesized compounds using triple-negative breast cancer MDA-MB-231 and glioblastoma U-87 cell lines by MTT assay revealed that hydrazones **15** and **16**, bearing two pyridine rings in their molecules, and the 5-chloropyridine derivative **21**, bearing a 4-methylthiobenzylidene moiety, possessed significant activity, and **21** was the most active one (an IC_50_ value of 39.2 ± 1.7 μM) against the MDA-MD-231. The results of the in silico molecular modeling suggested that these three most active hydrazones, **15**, **16** and **21**, were probably acting on the mitogen-activated protein kinase pathway through the inhibition of BRAF and MEK serine–threonine protein kinases. Hydrazone **16**, bearing two pyridine rings in its molecule, and the 5-chloropyridine derivative **21**, bearing a 4-methylthiobenzylidene moiety, were identified as compounds with significant antimicrobial and anticancer activity. They simultaneously possessed high antibacterial activity against the test culture *M. luteum* (MIC = 3.9 µg/mL) and antifungal activity against the strain *C. tenuis* (MIC = 1.9 µg/mL) as well as anticancer properties against the triple-negative breast cancer MDA-MB-231 and glioblastoma U-87 cells. Therefore, they could be promising candidates for the further investigation of dual-action agents.

## Figures and Tables

**Table 1:** The antimicrobial activity of compounds **5**–**21** determined by the diffusion assay.

Compound	Concentration, %	Diameter of Inhibition Zones of Microorganism Growth, mm				
		*E. coli*	*S. aureus*	*M. luteum*	*C. tenuis*	*A. niger*
**5**	0.5	0	0	12.0	13.0	10.0
	0.1	0	0	8.0	9.0	8.0
**6**	0.5	0	0	18.0	15.0	11.0
	0.1	0	0	0	10.0	8.0
**7**	0.5	0	0	10.0	11.0	10.0
	0.1	0	0	0	8.0	8.0
**8**	0.5	0	0	13.0	8.0	11.0
	0.1	0	0	10.0	0	8.0
**10**	0.5	0	0	13.0	13.0	9.0
	0.1	0	0	10.0	8.0	0
**11**	0.5	0	0	0	0	7.0
	0.1	0	0	0	0	0
**12**	0.5	0	0	15.0	15.4	7.0
	0.1	0	0	8.0	10.0	0
**13**	0.5	0	0	0	0	7.0
	0.1	0	0	0	0	0
**14**	0.5	0	0	0	0	7.0
	0.1	0	0	0	0	0
**15**	0.5	0	0	0	0	7.0
	0.1	0	0	0	0	0
**16**	0.5	0	14.4	12.4	14.0	10.0
	0.1	0	0	0	0	7.0
**18**	0.5	0	0	12.0	12.0	12.0
	0.1	0	0	0	0	0
**19**	0.5	0	0	9.0	0	0
	0.1	0	0	0	0	0
**20**	0.5	0	0	10.0	0	0
	0.1	0	0	0	0	0
**21**	0.5	0	0	13.0	12.0	8.0
	0.1	0	0	10.0	9.0	0
Control *	0.5	14.0	15.0	18.0	19.0	20.0

* Vancomycin (an antibacterial substance) and nystatin (an antifungal substance) were used as the controls.

**Table 2:** The minimal inhibitory concentrations of compounds **5**–**21** determined by the serial dilution method.

Compound/Microorganism Cultures	MIC, µg/mL				
	*E. coli*	*S. aureus*	*M. luteum*	*C. tenuis*	*A. niger*
**5**	+	+	7.8	1.9	500.0
**6**	125.0	250.0	7.8	0.9	500.0
**7**	+	+	7.8	0.9	500.0
**8**	+	+	3.9	1.9	500.0
**10**	+	+	3.9	3.9	250.0
**11**	+	+	+	500.0	31.2
**12**	+	+	15.6	15.6	31.2
**13**	+	+	31.2	31.2	125.0
**14**	+	+	31.2	31.2	125.0
**15**	+	+	250.0	+	500.0
**16**	+	250.0	3.9	1.9	500.0
**18**	+	+	62.5	7.8	31.2
**19**	+	+	62.5	+	500.0
**20**	500.0	+	31.2	62.5	31.2
**21**	+	+	3.9	3.9	62.5
Control *	3.9	7.8	7.8	15.6	3.9

+ growth of the microorganism was observed at the studied concentrations; * vancomycin (an antibacterial substance) and nystatin (an antifungal substance) were used as the controls.

**Table 3:** The docking score values for the binding of the **15**, **16** and **21** ligands with the serine–threonine protein kinases.

Ligand	Serine–Threonine Protein Kinases							
	BRAF (PDB: 1UWH)		MEK (PDB: 4U7Z)		BRAF (PDB: 5VAM)		BRAF (PDB: 4G9C)	
	Docking Score	Glide Emodel	Docking Score	Glide Emodel	Docking Score	Glide Emodel	Docking Score	Glide Emodel
	kcal/mol	kcal/mol	kcal/mol	kcal/mol	kcal/mol	kcal/mol	kcal/mol	kcal/mol
**15**	−9.648	−75.423	−7.766	−90.238	−9.126	−64.289	−9.529	−76.868
**16**	−9.574	−74.545	−7.384	−103.848	−9.380	−81.996	−9.624	−79.224
**21**	−10.087	−85.697	−9.103	−115.335	−10.122	−87.780	−9.932	−85.912
Dasatinib	−10.428	−101.336	−8.052	−104.318	−8.593	−87.010	−9.956	−94.452
Sorafenib	−10.093	−122.715	−6.406	−88.934	−10.349	−98.181	−10.676	−109.879
Vemurafenib	−11.313	−96.824	−7.275	−89.902	−10.285	−94.962	−10.136	−90.640

## Data Availability

All research data are provided in the current article.

## Supplementary Materials

The following supporting information can be downloaded at: https://www.mdpi.com/article/10.3390/biom14121529/s1, Figures S1–S50: ^1^H NMR, ^13^C NMR, and HRMS of compounds **5**–**21**.
