# Supplementary material for: Novel Antimicrobial and Antitumor Agents Bearing Pyridine-1,2,4-triazole-3-thione-hydrazone Scaffold: Synthesis, Biological Evaluation, and Molecular Docking Investigation
Source: Biomolecules. 2024 Nov 28;14(12):1529. doi: 10.3390/biom14121529 (PMC11673677; doi:10.3390/biom14121529)
Supplement: Supplementary file 1 [file biomolecules-14-01529-s001.zip › biomolecules-3307850-supplementary.pdf]

Supplementary Materials

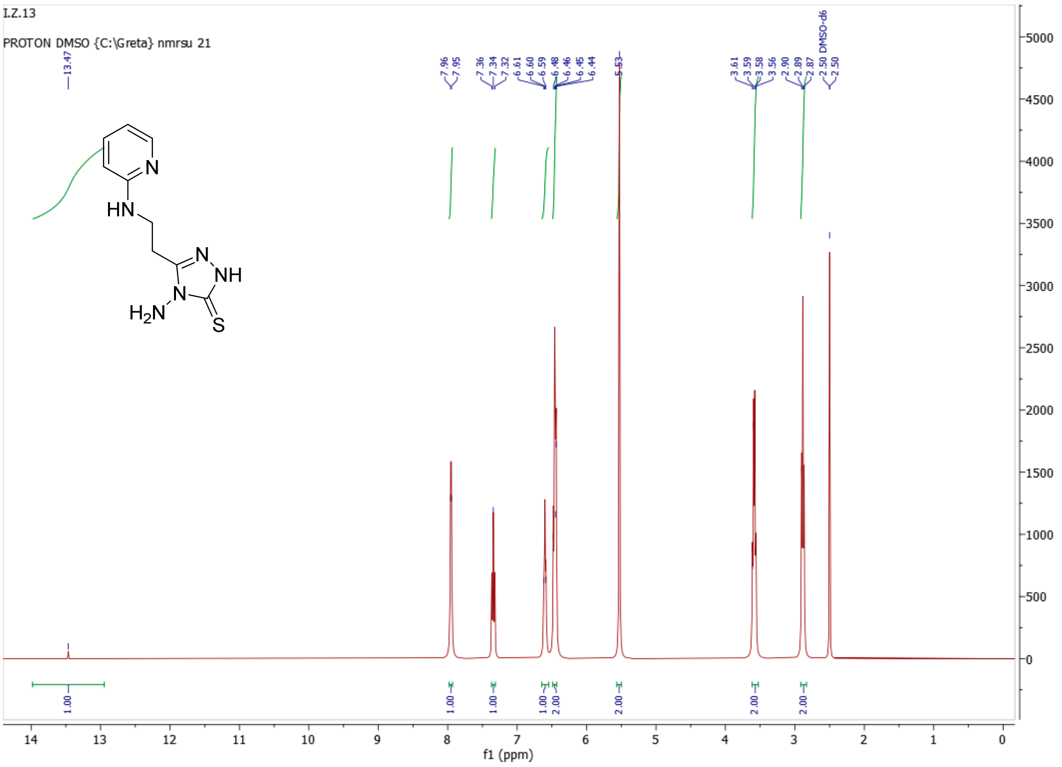

Figure S1. ^{1}H NMR (400 MHz, DMSO-d_6) spectrum of **5**.

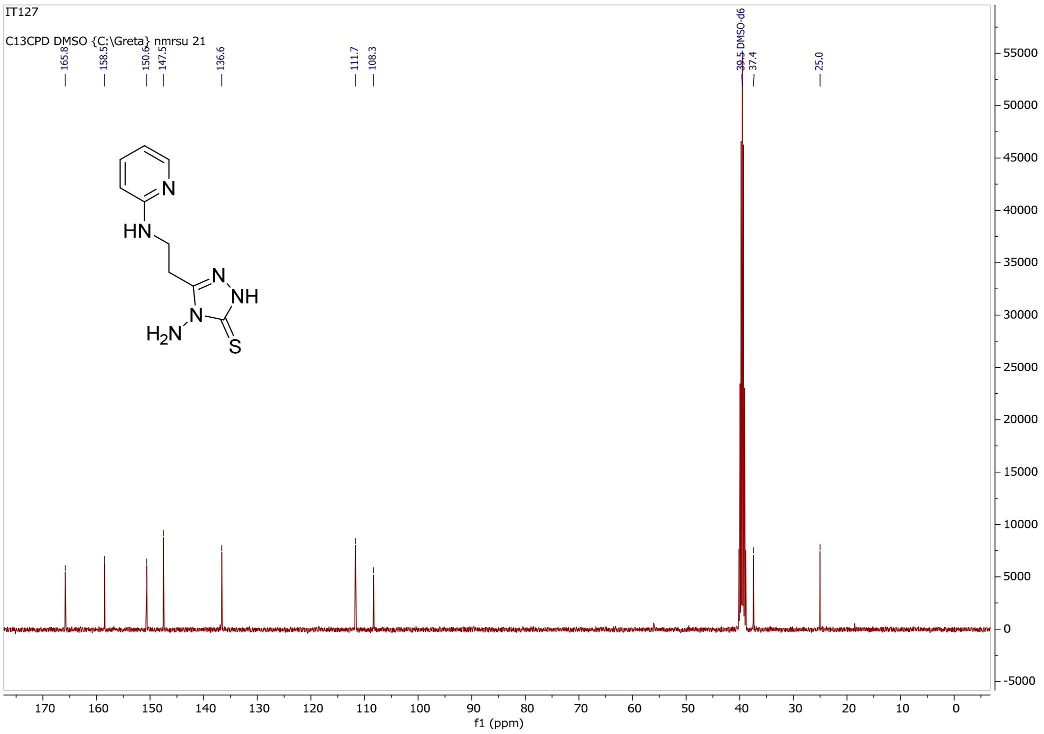

Figure S2. ^{13}C NMR (101 MHz, DMSO-d_6) spectrum of **5.**

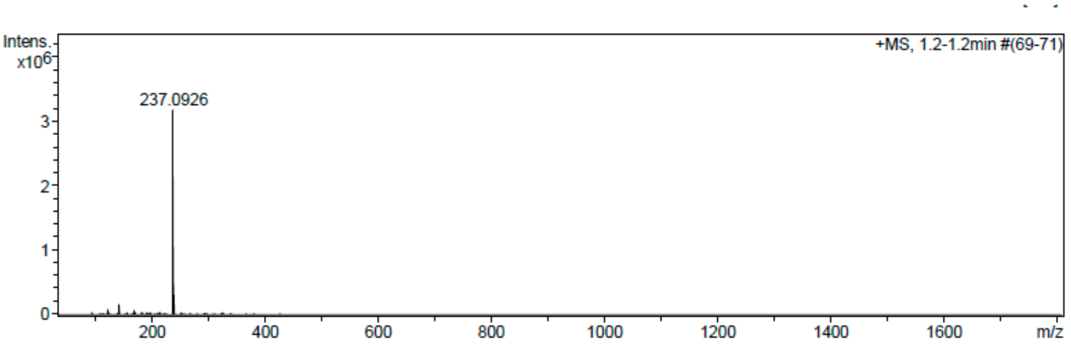

Meas. m/z	#	Formula	Score	m/z	err [ppm]	Mean err [ppm]	mSigma	rdb	e⁻ Conf	N-Rule
237.0926	1	C 13 H 17 O 2 S	70.15	237.0944	7.4	8.5	1.0	5.5	even	ok
	2	C 9 H 13 N 6 S	100.00	237.0917	-3.9	-2.9	10.3	6.5	even	ok
	3	C 16 H 13 O 2	55.77	237.0910	-6.8	-5.8	18.3	10.5	even	ok

Figure S3. HRMS spectrum of **5.**

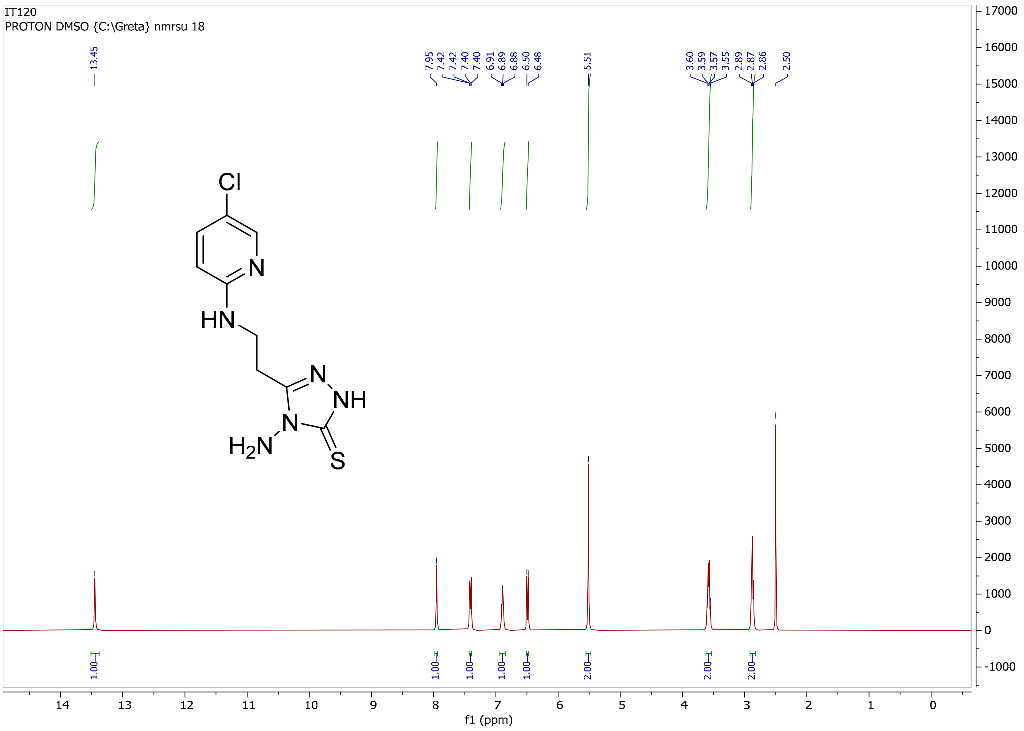

Figure S4. ^{1}H NMR (400 MHz, DMSO-d_6) spectrum of **6.**

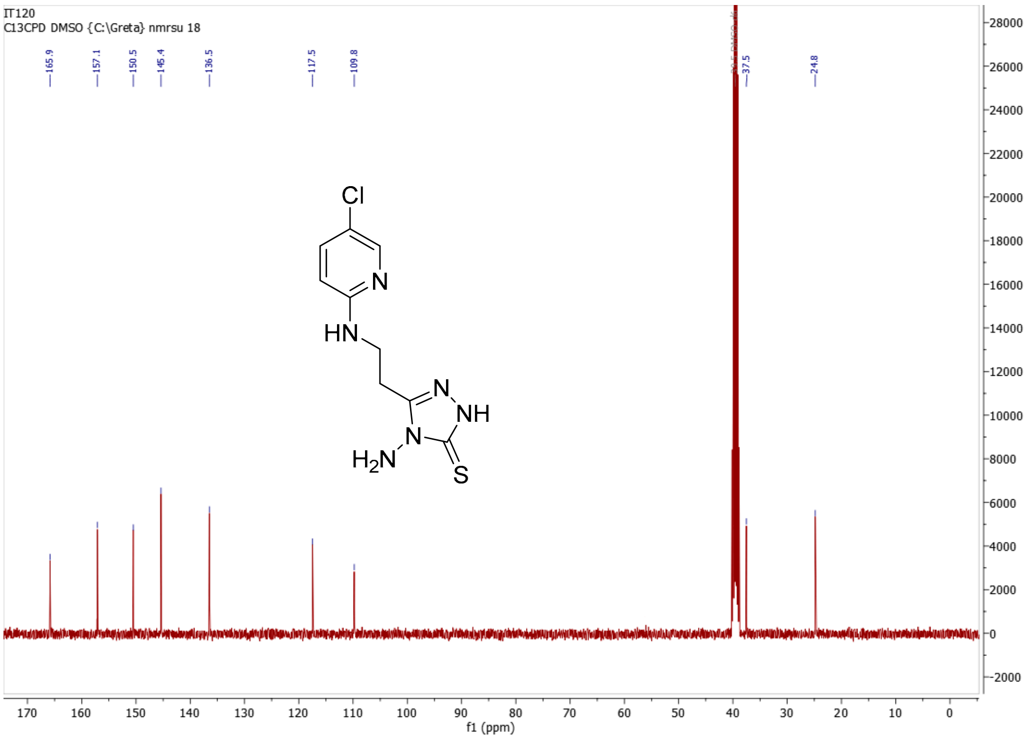

Figure S5. ^{13}C NMR (101 MHz, DMSO-d_6) spectrum of **6.**

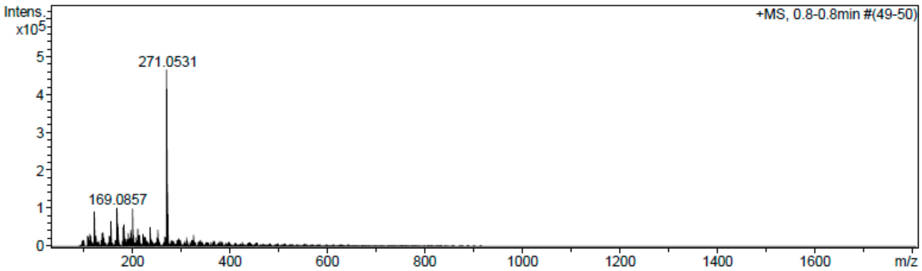

Meas. m/z	#	Formula	Score	m/z	err [ppm]	Mean err [ppm]	mSigma	rdb	e⁻ Conf	N-Rule
271.0531	1	C 6 H 7 N 8 O 5	100.00	271.0534	1.0	1.4	7.3	7.5	even	ok
	2	C 8 H 16 Cl N 2 O 4 S	40.02	271.0514	-6.4	-5.7	10.8	1.5	even	ok
	3	C 7 H 3 N 12 O	42.64	271.0547	5.9	6.3	12.9	12.5	even	ok
	4	C 9 H 12 Cl N 6 S	77.41	271.0527	-1.5	-0.8	17.2	6.5	even	ok
	5	C 5 H 11 N 4 O 9	51.41	271.0521	-4.0	-3.5	20.1	2.5	even	ok
	6	C 16 H 12 Cl O 2	35.20	271.0520	-4.0	-3.2	35.6	10.5	even	ok
	7	C 22 H 7	7.31	271.0542	4.1	4.5	82.4	19.5	even	ok
	8	C 14 H 11 N 2 O 2 S	0.30	271.0536	1.6	1.3	148.9	10.5	even	ok

Figure S6. HRMS spectrum of **6.**

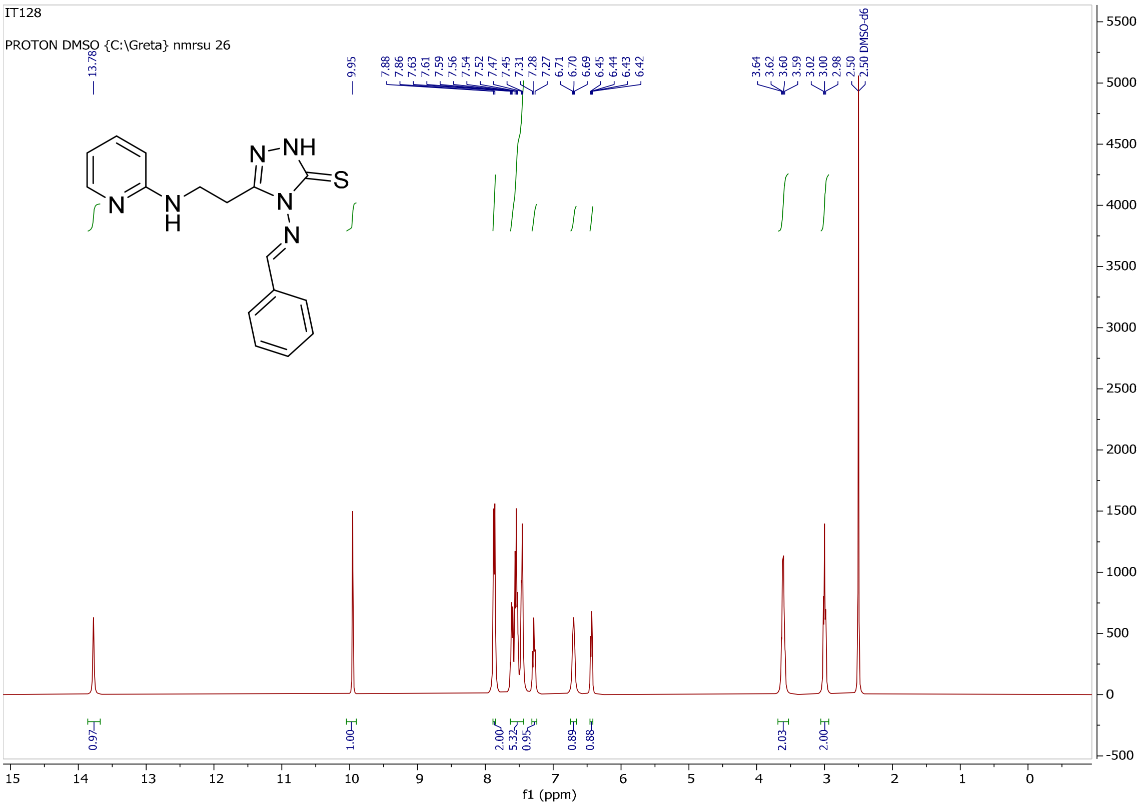

Figure S7. ^{1}H NMR (400 MHz, DMSO-d_6) spectrum of **7**.

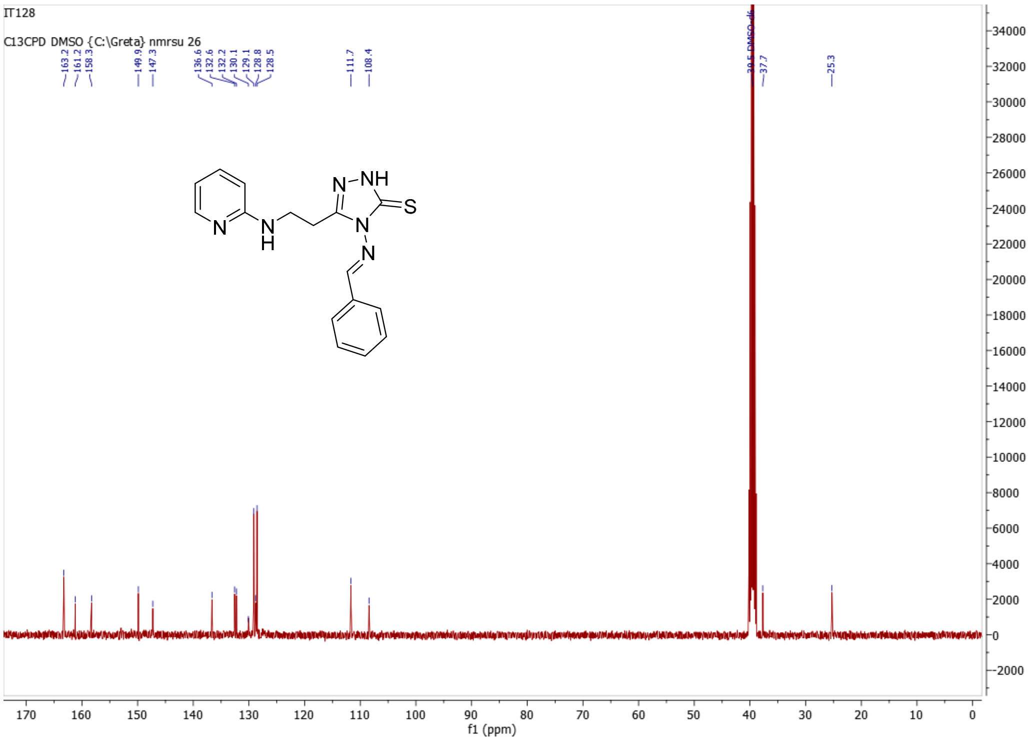

Figure S8. ^{13}C NMR (101 MHz, DMSO-d_6) spectrum of **7.**

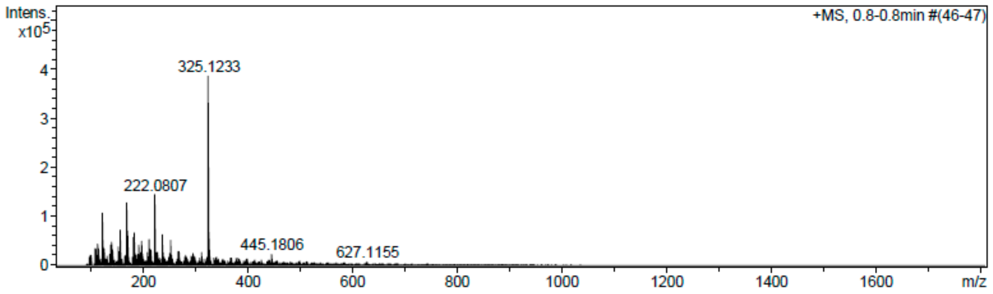

Meas. m/z	#	Formula	Score	m/z	err [ppm]	Mean err [ppm]	mSigma	rdb	e⁻ Conf	N-Rule
325.1233	1	C 15 H 21 N 2 O 4 S	55.54	325.1217	-5.1	-5.1	10.0	6.5	even	ok
	2	C 16 H 17 N 6 S	100.00	325.1230	-1.0	-1.0	18.9	11.5	even	ok
	3	C 11 H 21 N 2 O 9	62.98	325.1242	2.6	3.7	28.0	2.5	even	ok
	4	C 23 H 17 O 2	31.27	325.1223	-3.1	-2.8	50.4	15.5	even	ok

Figure S9. HRMS spectrum of **7.**

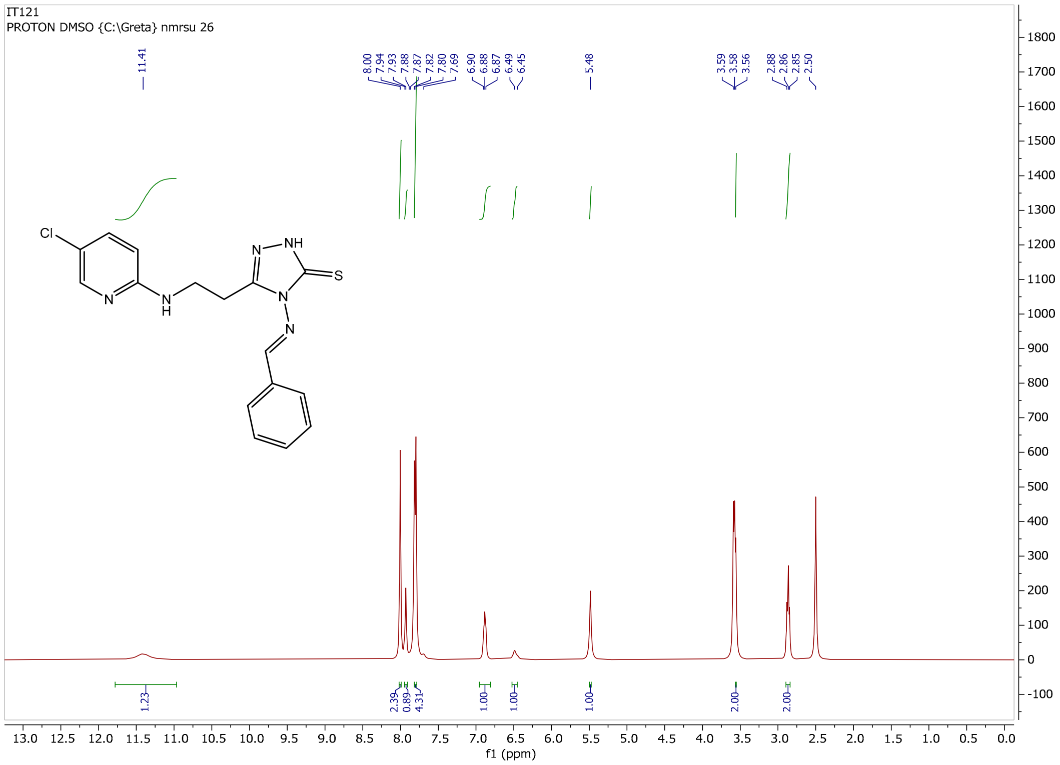

Figure S10. ^{1}H NMR (400 MHz, DMSO-d_6) spectrum of **8** .

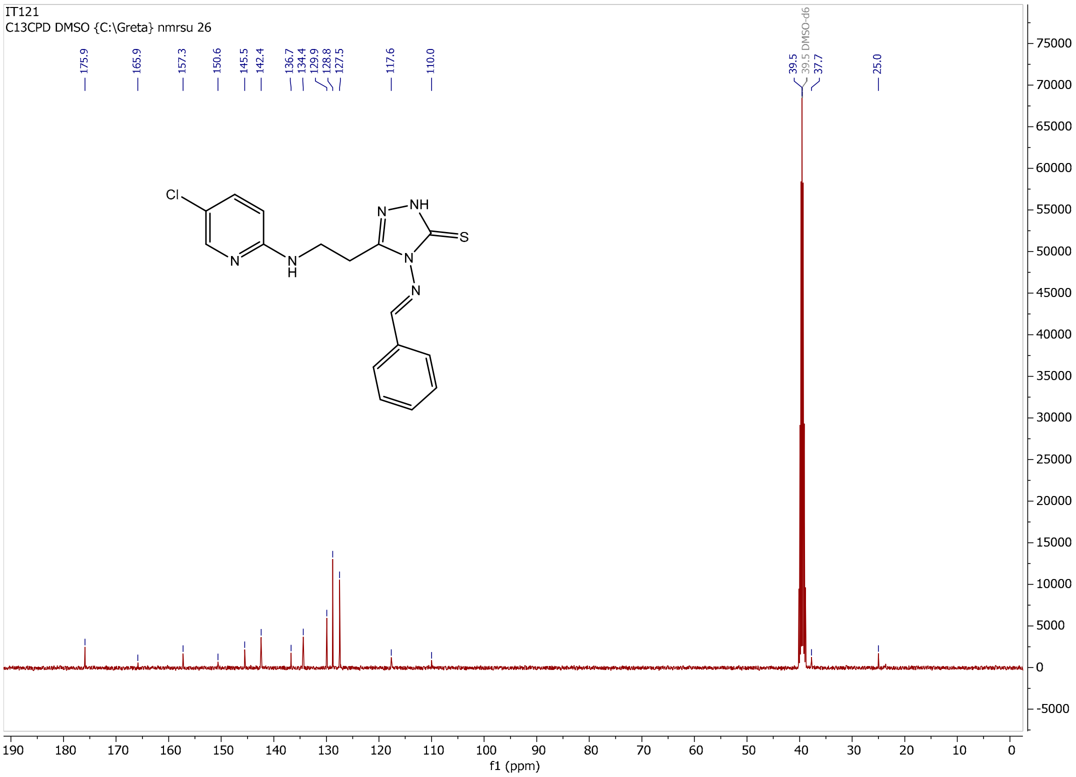

Figure S11. ^{13}C NMR (400 MHz, DMSO-*d*$_6$) spectrum of **8.**

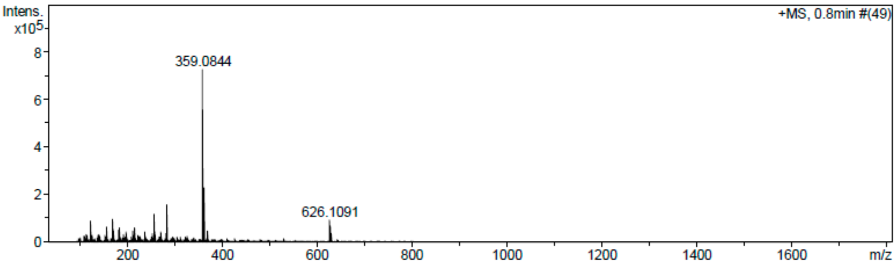

Meas. m/z	#	Formula	Score	m/z	err [ppm]	Mean err [ppm]	mSigma	rdb	e⁻ Conf	N-Rule
359.0844	1	C 8 H 12 Cl N 12 O 3	86.86	359.0838	-1.7	-1.3	14.5	8.5	even	ok
	2	C 13 H 11 N 8 O 5	100.00	359.0847	0.7	1.0	16.0	12.5	even	ok
	3	C 12 H 15 N 4 O 9	62.99	359.0834	-3.0	-2.7	17.5	7.5	even	ok
	4	C 11 H 20 Cl N 2 O 9	74.43	359.0852	2.1	2.7	18.8	2.5	even	ok
	5	C 14 H 7 N 12 O	38.91	359.0860	4.4	4.7	25.2	17.5	even	ok
	6	C 7 H 16 Cl N 8 O 7	29.96	359.0825	-5.4	-4.9	25.3	3.5	even	ok
	7	C 15 H 20 Cl N 2 O 4 S	27.48	359.0827	-4.9	-4.5	34.4	6.5	even	ok
	8	C 16 H 16 Cl N 6 S	53.98	359.0840	-1.2	-0.8	38.7	11.5	even	ok
	9	C 23 H 16 Cl O 2	29.93	359.0833	-3.1	-2.4	46.8	15.5	even	ok
	10	C 29 H 11	8.61	359.0855	3.0	3.3	82.5	24.5	even	ok
	11	C 21 H 15 N 2 O 2 S	1.24	359.0849	1.2	0.9	128.6	15.5	even	ok
	12	C 5 H 15 N 10 O 7 S	0.33	359.0840	-1.1	-1.6	150.4	3.5	even	ok
	13	C 6 H 11 N 14 O 3 S	0.23	359.0854	2.6	2.1	151.7	8.5	even	ok

Figure S12. HRMS spectrum of **8.**

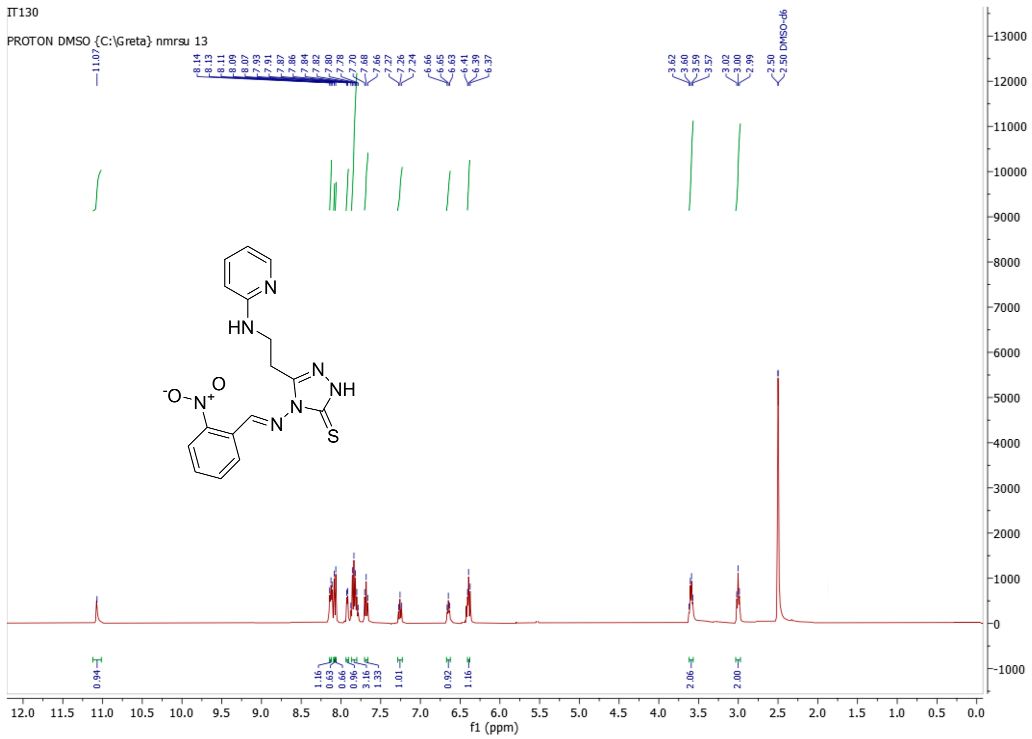

Figure S13. ^{1}H NMR (400 MHz, DMSO-d_6) spectrum of **9** .

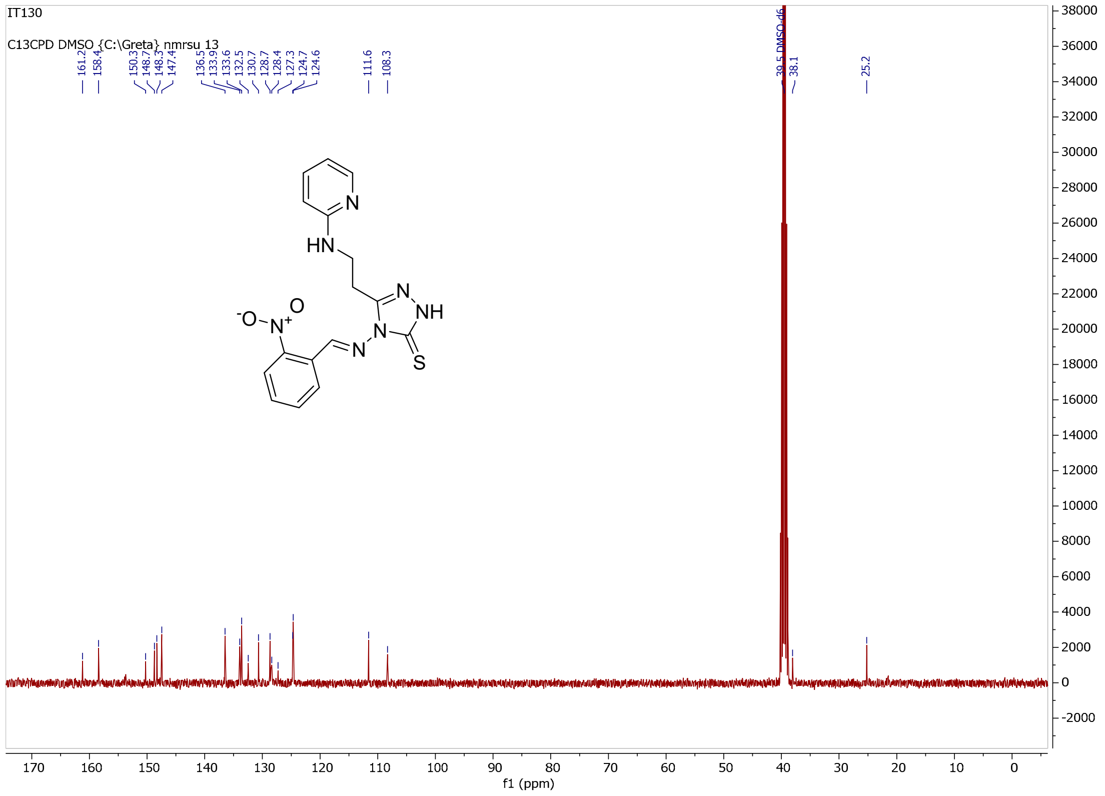

Figure S14. ^{13}C NMR (101 MHz, DMSO-d_6) spectrum of **9.**

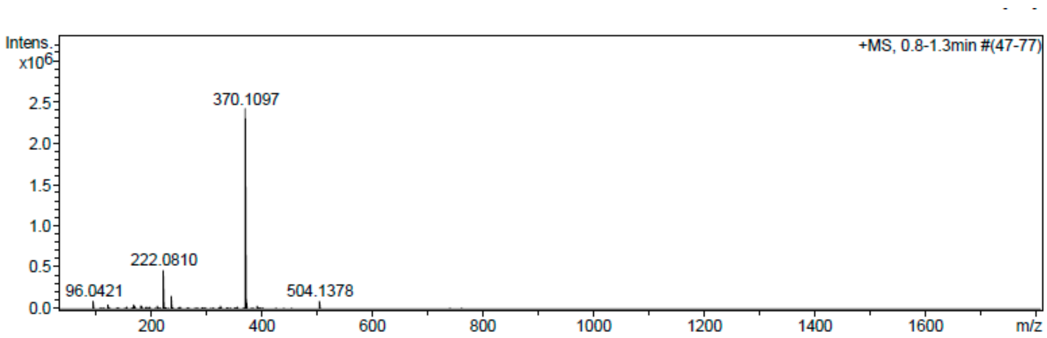

Meas. m/z	#	Formula	Score	m/z	err [ppm]	Mean err [ppm]	mSigma	rdb	e⁻ Conf	N-Rule
370.1097	1	C 24 H 12 N 5	100.00	370.1087	-2.7	-1.6	23.6	21.5	even	ok
	2	C 20 H 20 N O 4 S	74.76	370.1108	2.8	3.8	35.3	11.5	even	ok

Figure S15. HRMS spectrum of **9.**

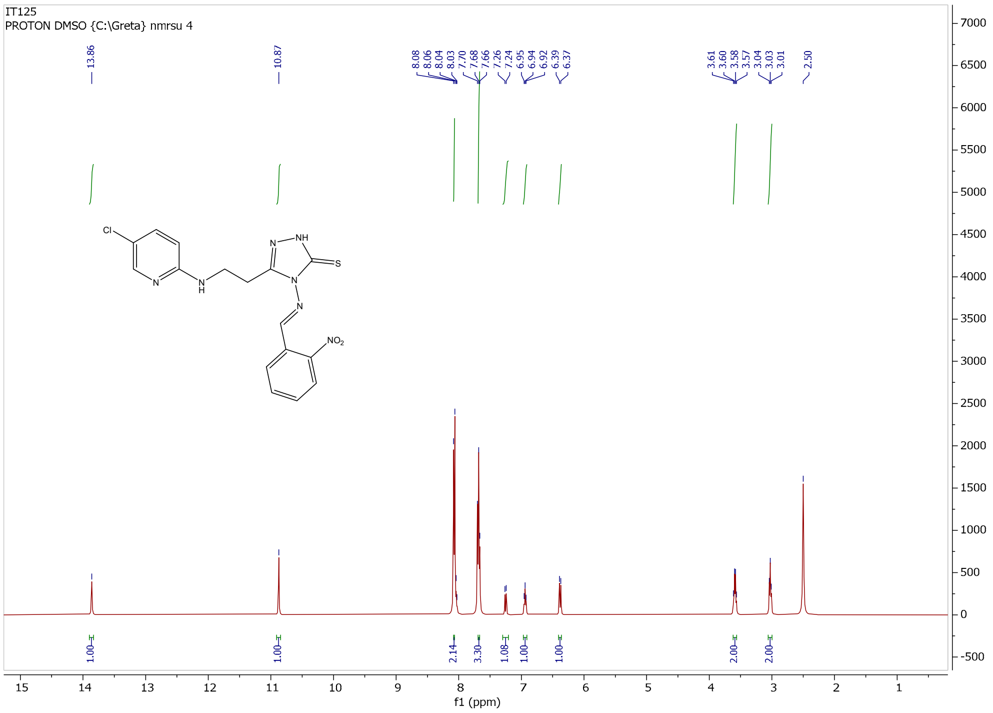

Figure S16. ^{1}H NMR (400 MHz, DMSO-d_6) spectrum of **10**.

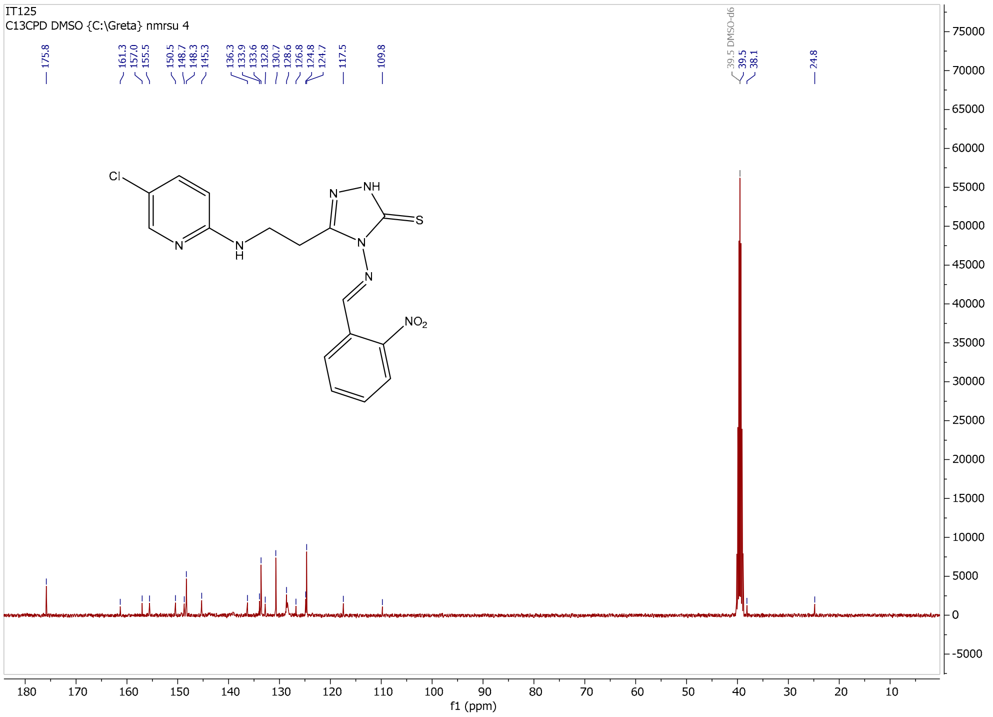

Figure S17. ^{13}C NMR (101 MHz, DMSO-d_6) spectrum of **10.**

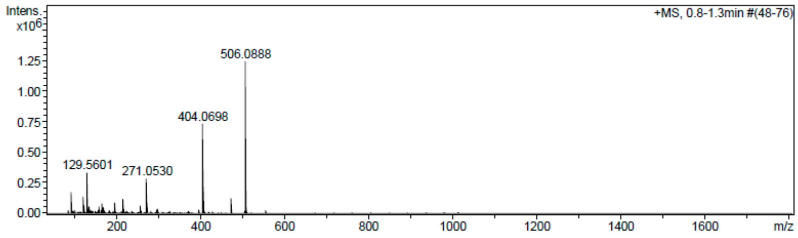

Meas. m/z	#	Formula	Score	m/z	err [ppm]	Mean err [ppm]	mSigma	rdb	e⁻ Conf	N-Rule
271.0530	1	C 6 H 7 N 8 O 5	100.00	271.0534	1.3	1.7	8.2	7.5	even	ok
	2	C 7 H 3 N 12 O	44.45	271.0547	6.2	6.6	10.6	12.5	even	ok
	3	C 8 H 16 Cl N 2 O 4 S	36.48	271.0514	-6.2	-5.4	20.7	1.5	even	ok
	4	C 5 H 11 N 4 O 9	54.30	271.0521	-3.7	-3.2	22.0	2.5	even	ok
	5	C 9 H 12 Cl N 6 S	73.99	271.0527	-1.2	-0.5	23.4	6.5	even	ok
	6	C 16 H 12 Cl O 2	40.74	271.0520	-3.7	-2.9	33.7	10.5	even	ok
	7	C 22 H 7	8.06	271.0542	4.3	4.9	80.2	19.5	even	ok
	8	C 14 H 11 N 2 O 2 S	0.63	271.0536	1.9	1.6	137.5	10.5	even	ok
404.0698	1	C 9 H 7 Cl N 17 O	95.63	404.0703	1.0	1.4	13.9	14.5	even	ok
	2	C 13 H 10 N 9 O 7	100.00	404.0698	-0.2	0.1	19.1	13.5	even	ok
	3	C 12 H 15 Cl N 7 O 7	38.85	404.0716	4.4	4.9	19.2	8.5	even	ok
	4	C 12 H 14 N 5 O 11	49.20	404.0684	-3.5	-3.1	19.4	8.5	even	ok

Figure S18. HRMS spectrum of **10.**

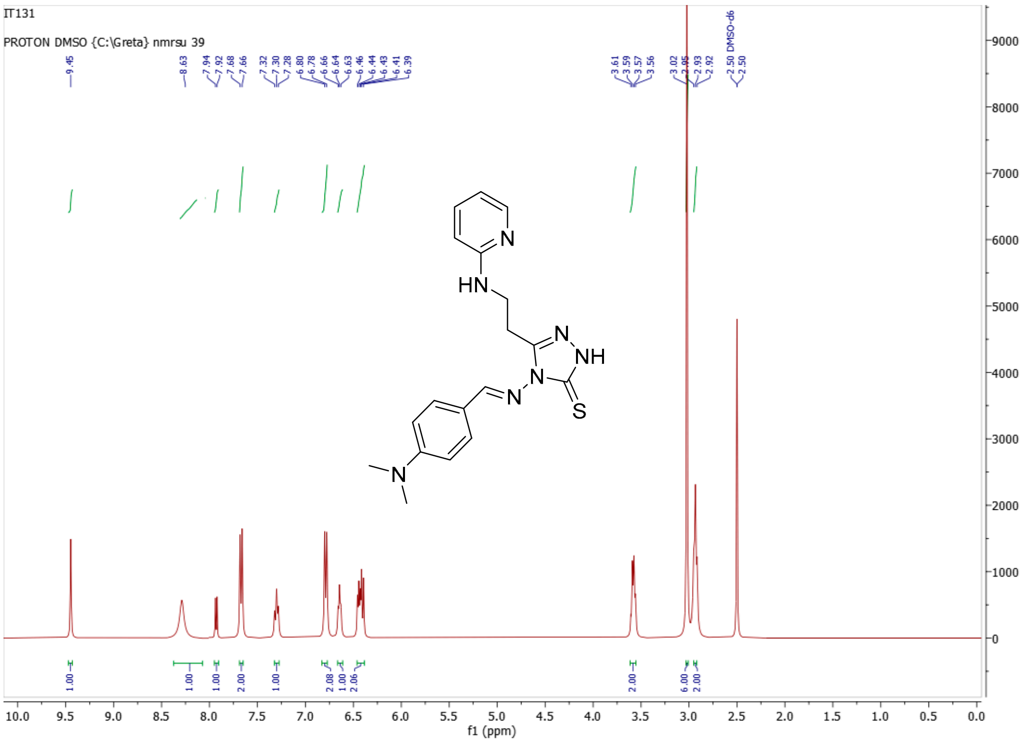

Figure S19. ^{1}H NMR (400 MHz, DMSO-d_6) spectrum of **11**.

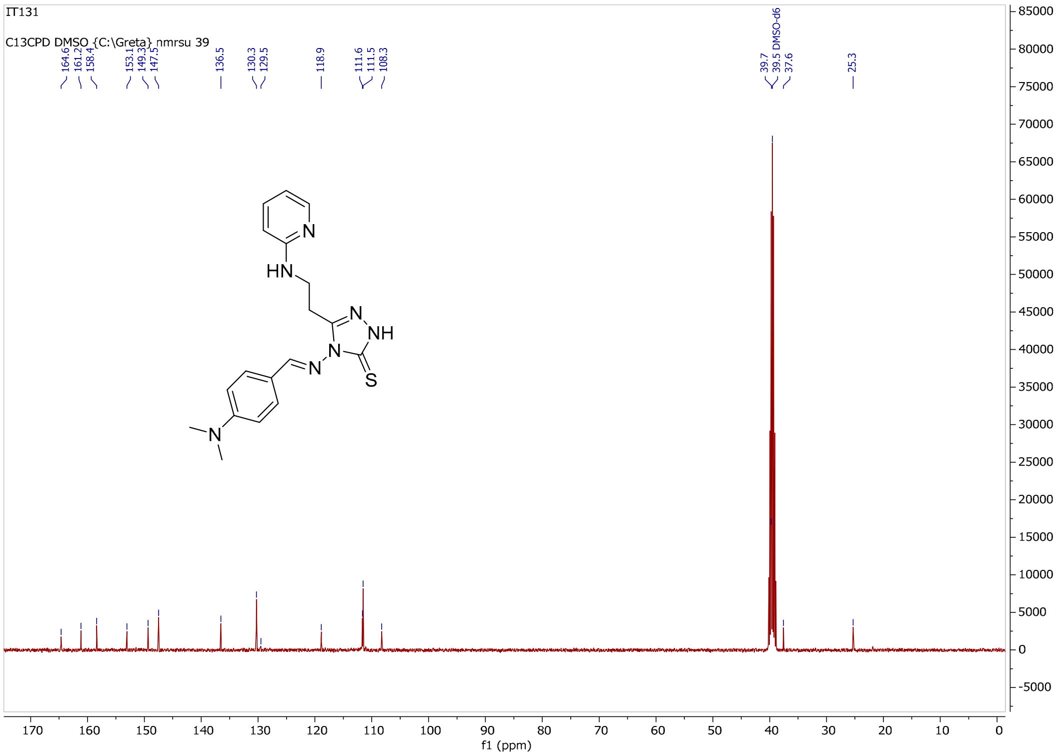

Figure S20. ^{13}C NMR (101 MHz, DMSO-d_6) spectrum of **11.**

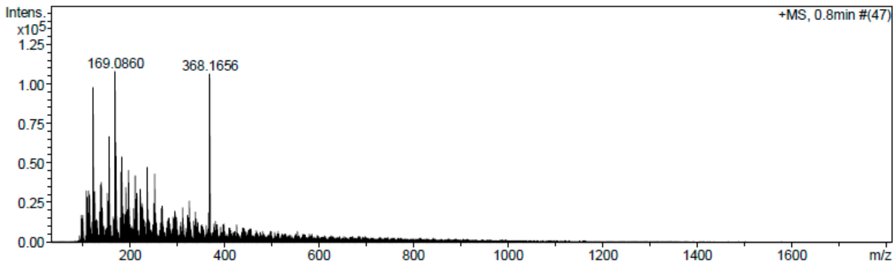

Meas. m/z	#	Formula	Score	m/z	err [ppm]	Mean err [ppm]	mSigma	rdb	e^- Conf	N-Rule
368.1656	1	C 18 H 22 N 7 S	100.00	368.1652	-1.1	-1.5	25.7	11.5	even	ok
	2	C 25 H 22 N O 2	66.02	368.1645	-3.0	-1.7	28.0	15.5	even	ok
	3	C 17 H 26 N 3 O 4 S	42.03	368.1639	-4.8	-5.1	29.1	6.5	even	ok

Figure S21. HRMS spectrum of **11.**

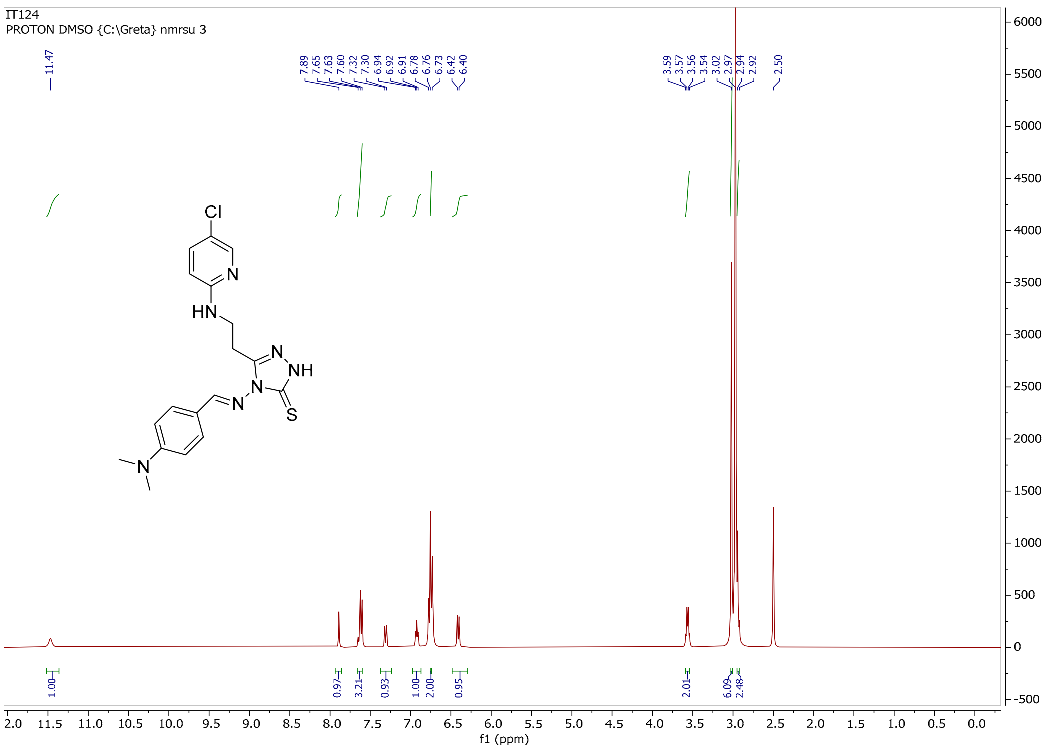

Figure S22. ^{1}H NMR (400 MHz, DMSO-d_6) spectrum of **12** .

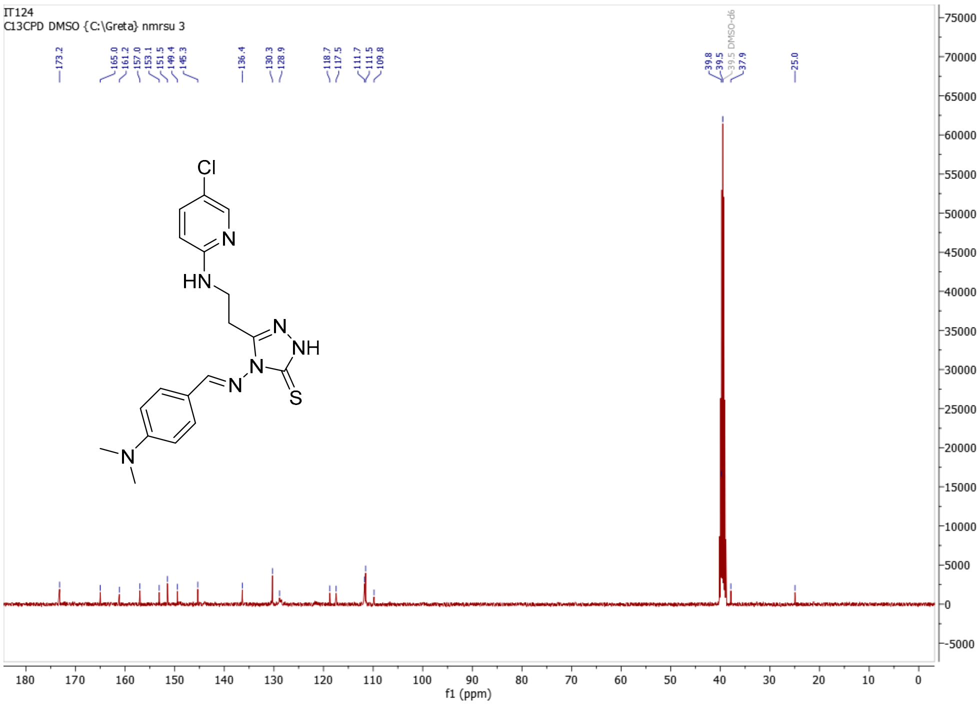

Figure S23. ^{13}C NMR (101 MHz, DMSO-d_6) spectrum of **12.**

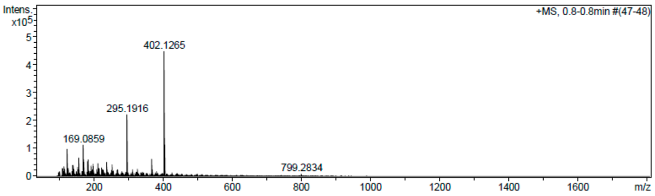

Meas. m/z	#	Formula	Score	m/z	err [ppm]	Mean err [ppm]	mSigma	rdb	e⁻ Conf	N-Rule
295.1916	1	C 17 H 27 O 4	75.06	295.1904	-4.1	-4.0	0.7	4.5	even	ok
	2	C 18 H 23 N 4	100.00	295.1917	0.4	0.5	15.6	9.5	even	ok
	3	C 10 H 27 N 6 O 2 S	51.17	295.1911	-1.8	-1.6	36.7	0.5	even	ok
	4	C 17 H 28 Cl N 2	0.10	295.1936	6.6	6.8	153.2	4.5	even	ok
402.1265	1	C 15 H 16 N 9 O 5	100.00	402.1269	0.9	1.2	16.2	12.5	even	ok
	2	C 16 H 12 N 13 O	42.18	402.1282	4.2	4.5	20.8	17.5	even	ok
	3	C 14 H 20 N 5 O 9	62.36	402.1256	-2.5	-2.1	22.7	7.5	even	ok
	4	C 10 H 17 Cl N 13 O 3	77.24	402.1260	-1.3	-0.8	24.5	8.5	even	ok
	5	C 13 H 25 Cl N 3 O 9	62.01	402.1274	2.1	2.7	26.9	2.5	even	ok
	6	C 9 H 21 Cl N 9 O 7	27.68	402.1247	-4.6	-4.0	33.5	3.5	even	ok
	7	C 17 H 25 Cl N 3 O 4 S	27.96	402.1249	-4.1	-3.7	38.1	6.5	even	ok
	8	C 18 H 21 Cl N 7 S	56.74	402.1262	-0.8	-0.4	40.2	11.5	even	ok
	9	C 25 H 21 Cl N O 2	36.10	402.1255	-2.5	-1.8	43.9	15.5	even	ok
	10	C 31 H 16 N	10.16	402.1277	2.9	3.3	78.2	24.5	even	ok
	11	C 23 H 20 N 3 O 2 S	1.83	402.1271	1.3	1.1	121.7	15.5	even	ok
	12	C 8 H 16 N 15 O 3 S	0.82	402.1276	2.6	2.2	131.2	8.5	even	ok
	13	C 4 H 12 N 21 O S	0.38	402.1249	-4.1	-6.9	137.2	9.5	even	ok
	14	C 7 H 20 N 11 O 7 S	0.45	402.1262	-0.8	-1.1	147.0	3.5	even	ok

Figure S24. HRMS spectrum of **12.**

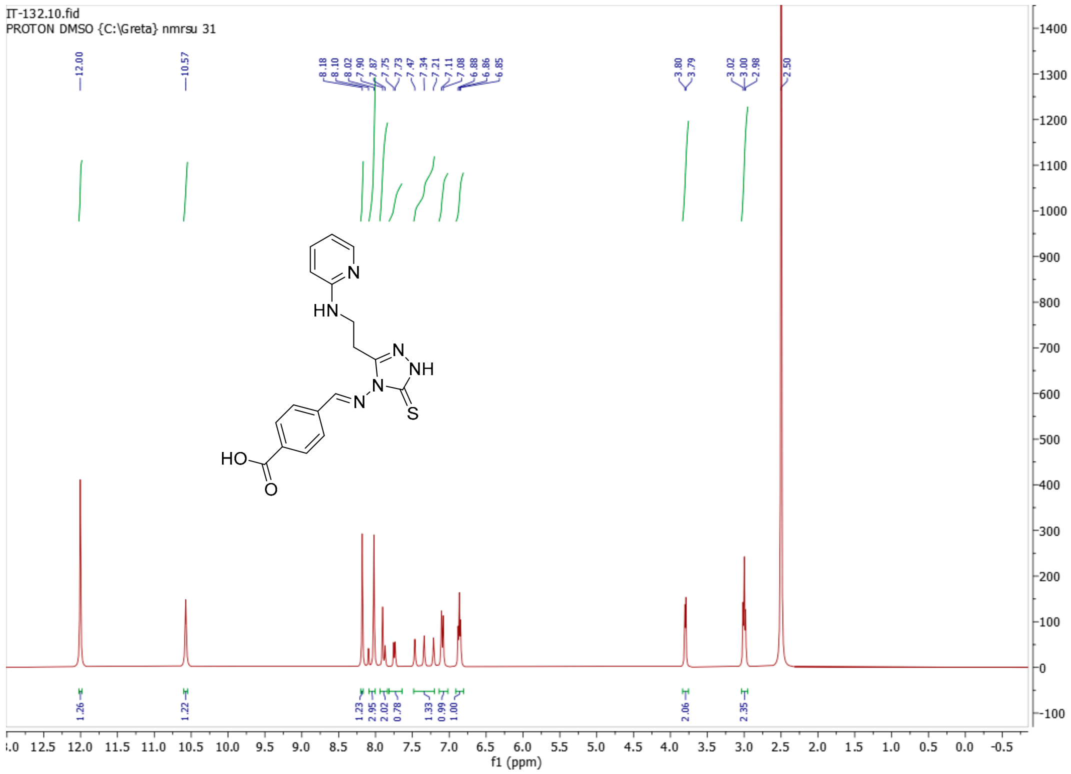

Figure S25. ^{1}H NMR (400 MHz, DMSO-d_6) spectrum of **13** .

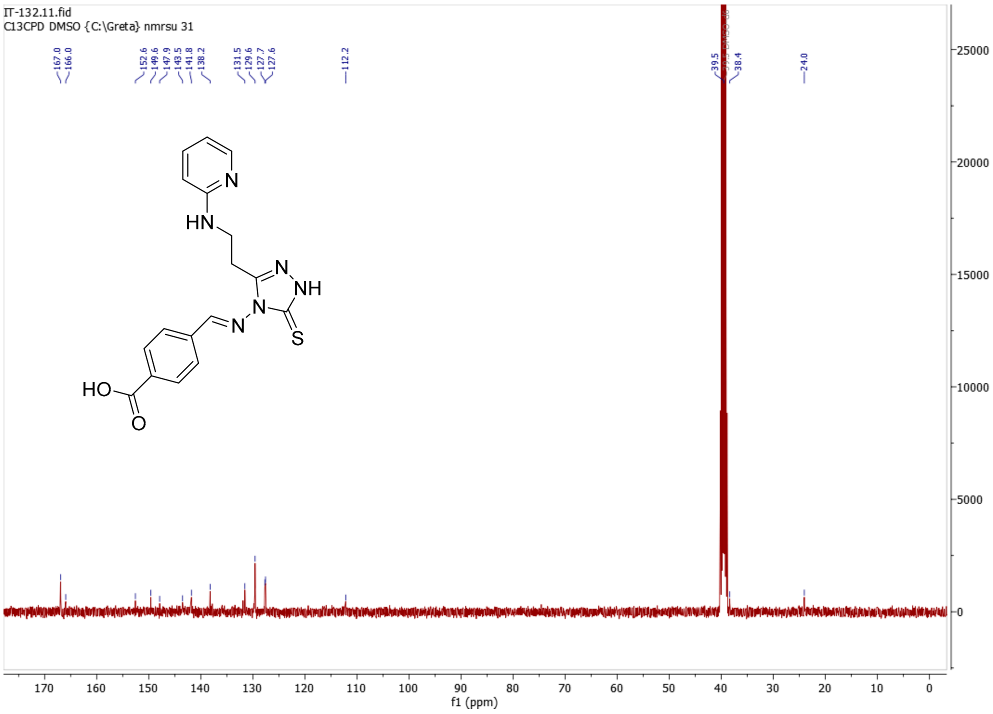

Figure S26. ^{13}C NMR (101 MHz, DMSO-d_6) spectrum of **13.**

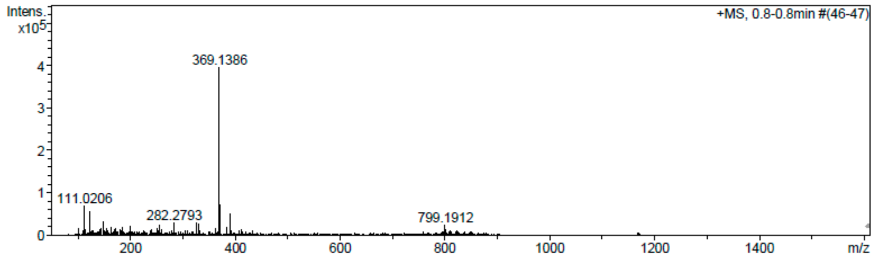

Meas. m/z	#	Formula	Score	m/z	err [ppm]	Mean err [ppm]	mSigma	rdb	e⁻ Conf	N-Rule
369.1386	1	$C_{15}H_{21}N_4O_7$	49.28	369.1405	5.0	5.8	8.4	7.5	even	ok
	2	$C_{14}H_{15}N_{11}O_2$	45.18	369.1405	5.0	5.9	13.2	13.0	odd	ok
	3	$C_{13}H_{19}N_7O_6$	100.00	369.1391	1.4	2.1	13.8	8.0	odd	ok
	4	$C_{12}H_{13}N_{14}O$	96.06	369.1391	1.3	2.3	16.0	13.5	even	ok
	5	$C_{14}H_{25}O_{11}$	94.14	369.1391	1.4	2.2	16.7	2.5	even	ok
	6	$C_{11}H_{17}N_{10}O_5$	74.27	369.1378	-2.3	-1.6	20.0	8.5	even	ok
	7	$C_{18}H_{25}O_6S$	35.03	369.1366	-5.4	-5.4	20.0	6.5	even	ok
	8	$C_{17}H_{19}N_7OS$	33.87	369.1366	-5.4	-5.5	21.4	12.0	odd	ok
	9	$C_{12}H_{23}N_3O_{10}$	69.12	369.1378	-2.3	-1.5	23.4	3.0	odd	ok
	10	$C_{19}H_{21}N_4O_2S$	68.80	369.1380	-1.8	-1.8	27.6	11.5	even	ok
	11	$C_{21}H_{23}NO_3S$	59.05	369.1393	1.9	1.9	33.3	11.0	odd	ok
	12	$C_{27}H_{17}N_2$	39.14	369.1386	-0.0	1.0	58.0	20.5	even	ok

Figure S27. HRMS spectrum of **13.**

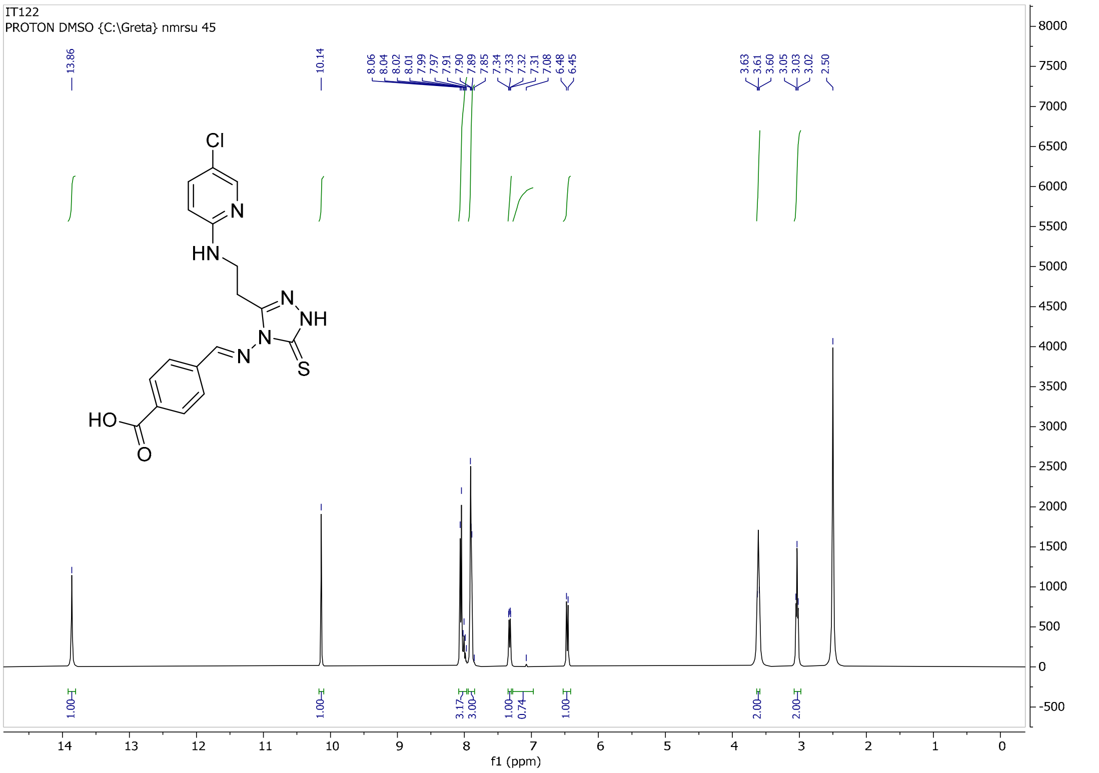

Figure S28. ^{1}H NMR (400 MHz, DMSO-d_6) spectrum of **14.**

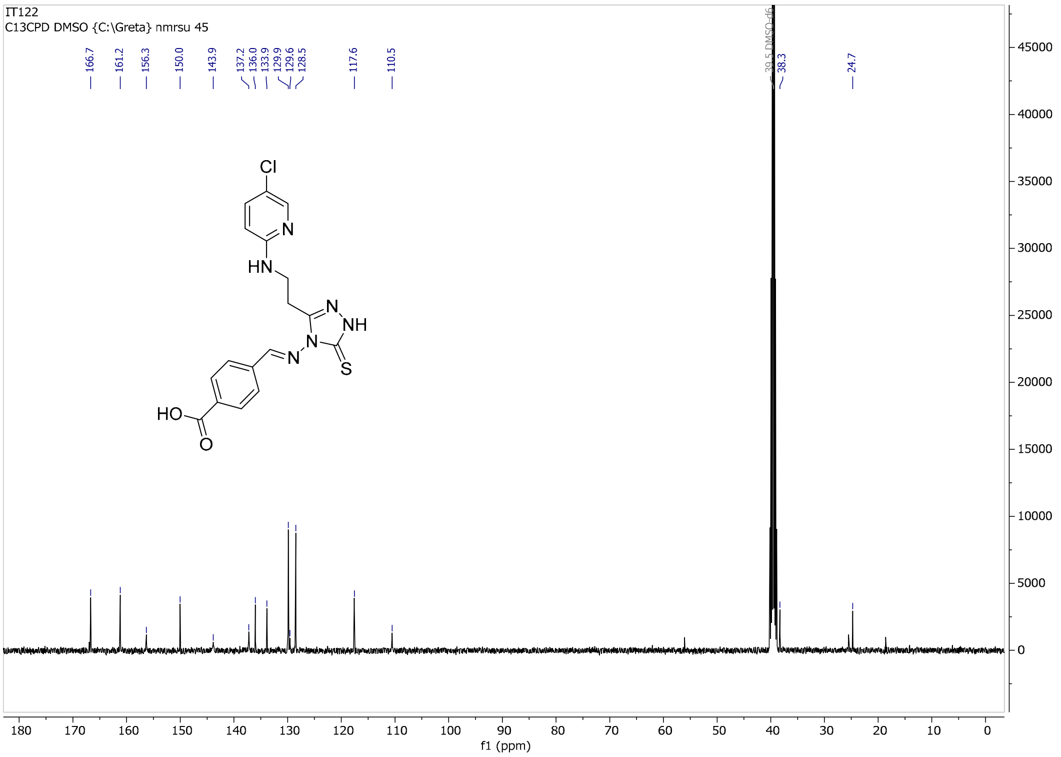

Figure S29. ^{13}C NMR (101 MHz, DMSO-d_6) spectrum of **14.**

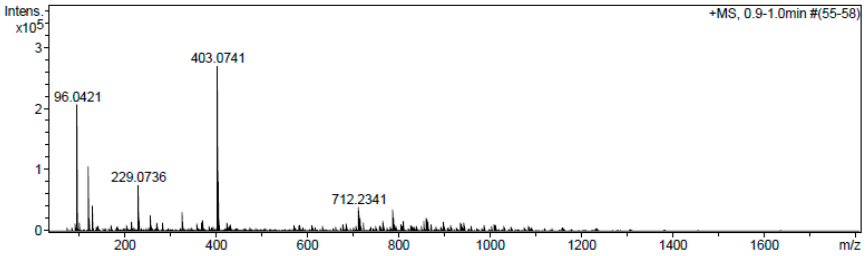

Meas. m/z	#	Formula	Score	m/z	err [ppm]	Mean err [ppm]	mSigma	rdb	e⁻ Conf	N-Rule
403.0741	1	C 17 H 16 Cl N 6 O 2 S	100.00	403.0738	-0.7	-0.2	14.1	12.5	even	ok
	2	C 16 H 20 Cl N 2 O 6 S	46.23	403.0725	-4.0	-3.5	15.0	7.5	even	ok
	3	C 15 H 7 N 12 O 3	42.64	403.0759	4.3	4.7	15.4	18.5	even	ok
	4	C 14 H 11 N 8 O 7	84.53	403.0745	1.0	1.4	20.0	13.5	even	ok
	5	C 10 H 8 Cl N 16 O	58.76	403.0750	2.2	2.7	25.4	14.5	even	ok
	6	C 24 H 16 Cl O 4	53.08	403.0732	-2.4	-1.5	27.4	16.5	even	ok
	7	C 12 H 20 Cl N 2 O 11	53.08	403.0750	2.2	2.9	29.5	3.5	even	ok
	8	C 13 H 15 N 4 O 11	50.16	403.0732	-2.4	-1.9	30.3	8.5	even	ok
	9	C 9 H 12 Cl N 12 O 5	57.57	403.0737	-1.2	-0.6	34.7	9.5	even	ok
	10	C 25 H 12 Cl N 4	53.63	403.0745	0.9	1.7	39.2	21.5	even	ok
	11	C 8 H 16 Cl N 8 O 9	21.12	403.0723	-4.5	-3.8	42.3	4.5	even	ok
	12	C 26 H 7 N 6	12.86	403.0727	-3.6	-3.2	65.4	26.5	even	ok
	13	C 30 H 11 O 2	13.36	403.0754	3.0	3.5	68.5	25.5	even	ok

Figure S30. HRMS spectrum of **14.**

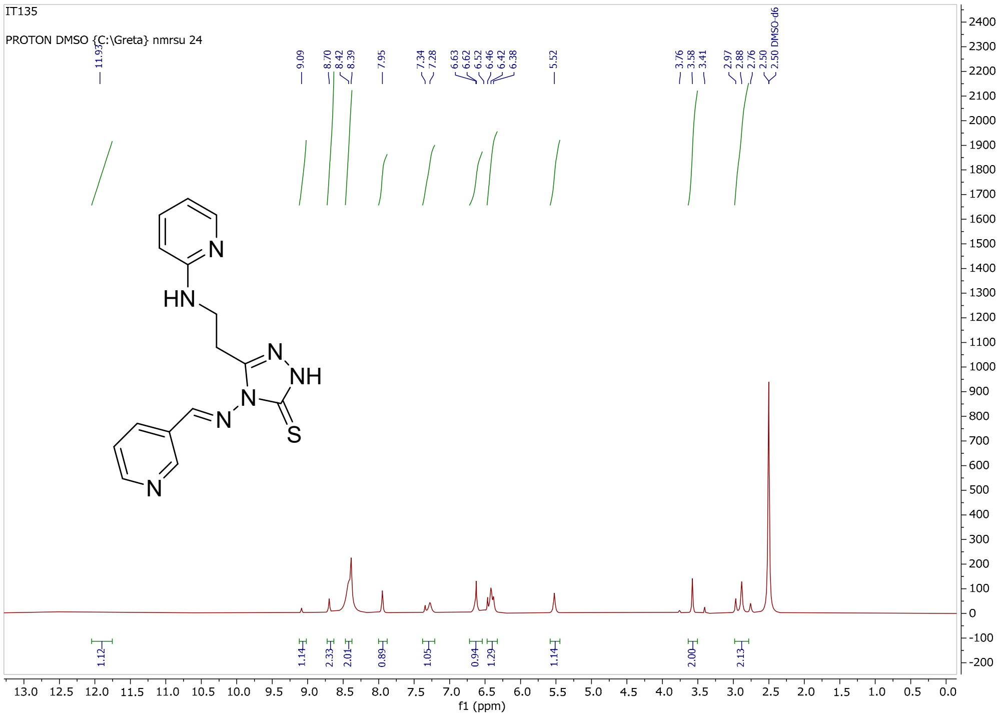

Figure S31. ^{1}H NMR (400 MHz, DMSO-d_6) spectrum of **15** .

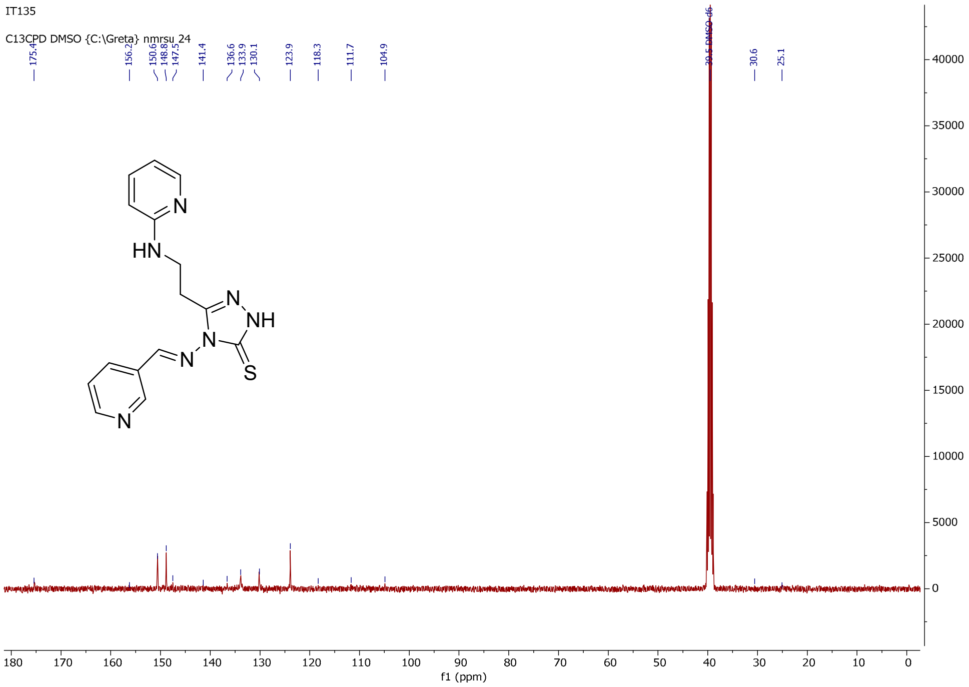

Figure S32. ^{13}C NMR (101 MHz, DMSO-d_6) spectrum of **15.**

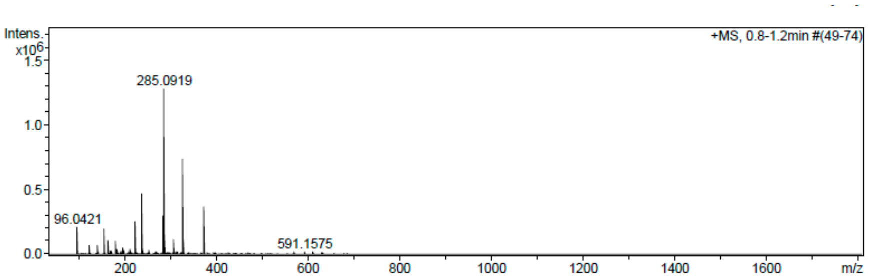

Meas. m/z	#	Formula	Score	m/z	err [ppm]	Mean err [ppm]	mSigma	rdb	e⁻ Conf	N-Rule
285.0919	1	C 5 H 9 N 12 O 3	100.00	285.0915	-1.4	-1.0	13.2	7.5	even	ok
	2	C 8 H 17 N 2 O 9	66.96	285.0929	3.4	3.8	18.5	1.5	even	ok
	3	C 12 H 17 N 2 O 4 S	45.89	285.0904	-5.4	-5.0	19.4	5.5	even	ok
	4	C 13 H 13 N 6 S	78.00	285.0917	-0.7	-0.4	28.4	10.5	even	ok
	5	C 20 H 13 O 2	24.14	285.0910	-3.1	-2.7	57.9	14.5	even	ok
326.1183	1	C 14 H 20 N 3 O 4 S	58.21	326.1169	-4.3	-4.1	14.4	6.5	even	ok
	2	C 15 H 16 N 7 S	100.00	326.1182	-0.2	0.0	22.2	11.5	even	ok
	3	C 10 H 20 N 3 O 9	58.29	326.1194	3.3	4.5	24.0	2.5	even	ok
	4	C 22 H 16 N O 2	32.90	326.1176	-2.3	-1.9	52.0	15.5	even	ok

Figure S33. HRMS spectrum of **15.**

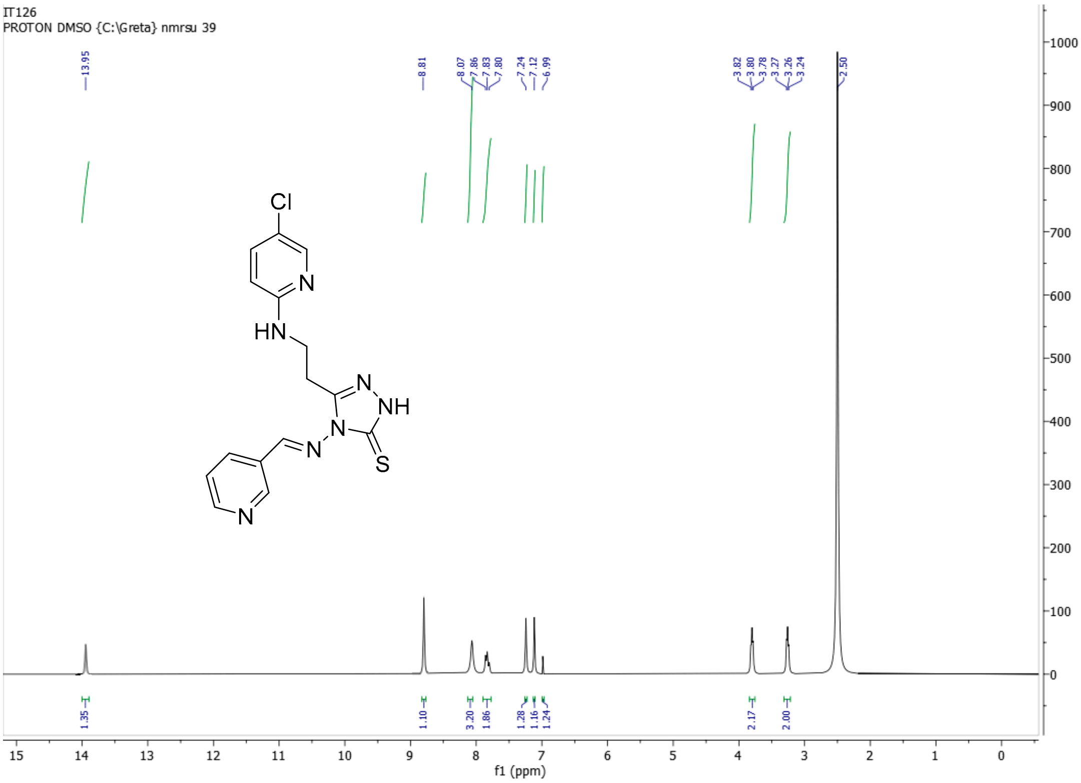

Figure S34. ^{1}H NMR (400 MHz, DMSO-d_6) spectrum of **16** .

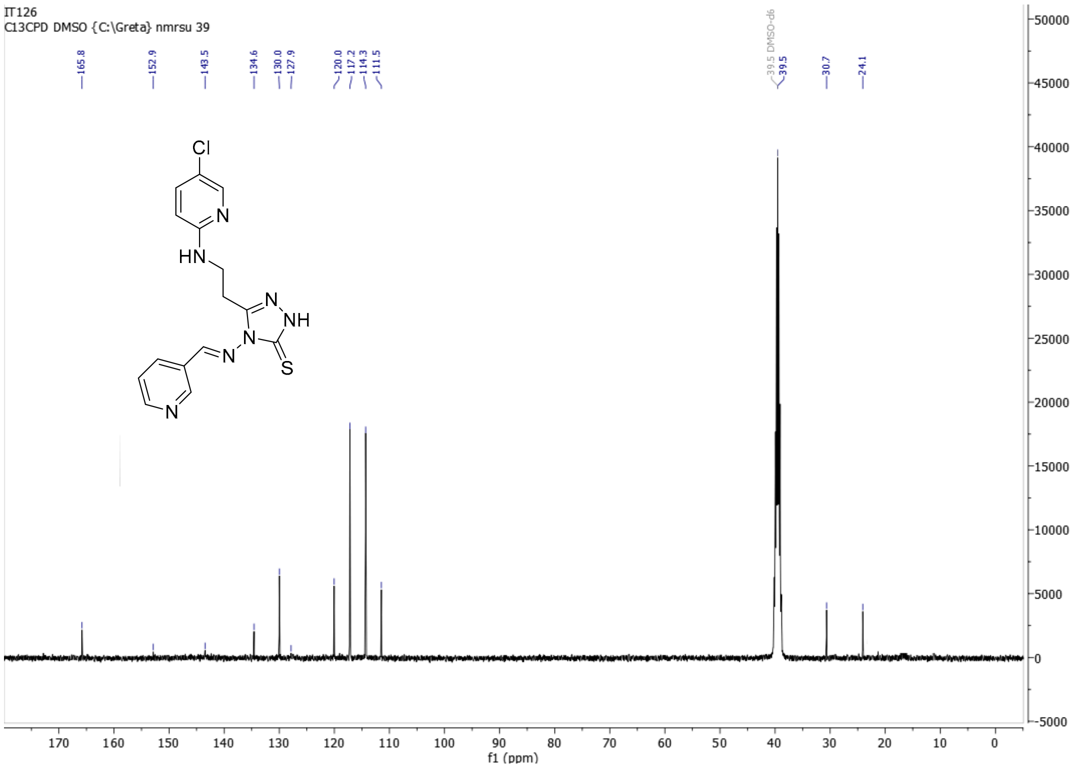

Figure S35. ^{13}C NMR (101 MHz, DMSO-d_6) spectrum of **16.**

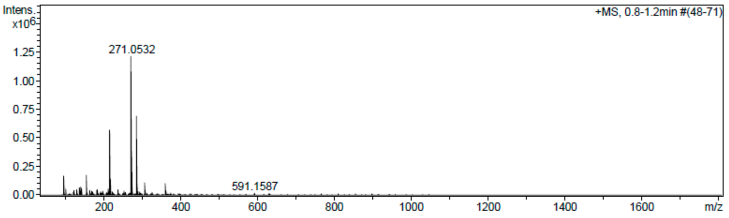

Meas. m/z	#	Formula	Score	m/z	err [ppm]	Mean err [ppm]	mSigma	rdb	e⁻ Conf	N-Rule
215.0585	1	C 9 H 12 Cl N 2 O 2	100.00	215.0582	-1.5	-1.2	32.4	4.5	even	ok
	2	C 15 H 7 N 2	22.79	215.0604	8.7	8.8	52.7	13.5	even	ok
	3	C 6 H 15 O 6 S	3.05	215.0584	-0.6	-1.4	122.3	-0.5	even	ok
	4	C 7 H 11 N 4 O 2 S	1.36	215.0597	5.6	4.7	126.7	4.5	even	ok
	5	C 3 H 7 N 10 S	0.66	215.0570	-6.8	-8.0	136.2	5.5	even	ok
271.0532	1	C 6 H 7 N 8 O 5	100.00	271.0534	0.6	1.0	8.9	7.5	even	ok
	2	C 5 H 11 N 4 O 9	54.17	271.0521	-4.3	-3.9	14.6	2.5	even	ok
	3	C 7 H 3 N 12 O	38.61	271.0547	5.6	5.9	20.2	12.5	even	ok
	4	C 8 H 16 Cl N 2 O 4 S	26.78	271.0514	-6.8	-6.1	26.5	1.5	even	ok
	5	C 9 H 12 Cl N 6 S	53.07	271.0527	-1.8	-1.2	31.6	6.5	even	ok
	6	C 16 H 12 Cl O 2	27.77	271.0520	-4.4	-3.6	42.1	10.5	even	ok
	7	C 22 H 7	5.61	271.0542	3.7	4.1	89.6	19.5	even	ok
	8	C 14 H 11 N 2 O 2 S	0.75	271.0536	1.3	1.0	134.9	10.5	even	ok
285.0921	1	C 12 H 17 N 2 O 4 S	52.48	285.0904	-6.1	-5.7	11.8	5.5	even	ok
	2	C 13 H 13 N 6 S	100.00	285.0917	-1.4	-1.1	18.7	10.5	even	ok
	3	C 5 H 9 N 12 O 3	82.63	285.0915	-2.0	-1.7	23.4	7.5	even	ok
	4	C 8 H 17 N 2 O 9	68.18	285.0929	2.7	3.1	27.8	1.5	even	ok
	5	C 20 H 13 O 2	33.54	285.0910	-3.8	-3.4	47.7	14.5	even	ok
	6	C 15 H 14 Cl N 4	0.40	285.0902	-6.8	-6.3	134.4	10.5	even	ok
	7	C 12 H 18 Cl N 4 S	0.19	285.0935	5.0	5.5	151.7	5.5	even	ok
	8	C 11 H 22 Cl O 4 S	0.35	285.0922	0.3	0.9	153.4	0.5	even	ok
307.0738	1	C 10 H 11 N 8 O 2 S	59.26	307.0720	-5.9	-5.6	6.9	9.5	even	ok
	2	C 14 H 15 N 2 O 4 S	100.00	307.0747	2.9	3.2	10.2	8.5	even	ok
	3	C 6 H 11 N 8 O 7	61.18	307.0745	2.3	2.7	36.5	5.5	even	ok
	4	C 18 H 7 N 6	46.20	307.0727	-3.7	-3.3	37.7	18.5	even	ok
	5	C 3 H 3 N 18 O	50.67	307.0732	-2.1	-3.8	44.4	11.5	even	ok
	6	C 5 H 15 N 4 O 11	43.74	307.0732	-2.1	-1.6	49.5	0.5	even	ok
	7	C 22 H 11 O 2	24.73	307.0754	5.0	5.5	50.9	17.5	even	ok
	8	C 16 H 16 Cl O 4	1.09	307.0732	-2.1	-1.6	132.4	8.5	even	ok
	9	C 17 H 12 Cl N 4	1.06	307.0745	2.2	2.7	132.6	13.5	even	ok
	10	C 9 H 16 Cl N 6 O 2 S	0.58	307.0738	0.1	0.5	147.3	4.5	even	ok
	11	C 8 H 20 Cl N 2 O 6 S	0.26	307.0725	-4.3	-3.8	149.2	-0.5	even	ok
360.0795	1	C 13 H 6 N 13 O	47.41	360.0813	5.0	5.2	11.1	17.5	even	ok
	2	C 12 H 10 N 9 O 5	96.41	360.0799	1.3	1.6	15.4	12.5	even	ok
	3	C 14 H 19 Cl N 3 O 4 S	47.89	360.0779	-4.3	-3.9	18.6	6.5	even	ok
	4	C 15 H 15 Cl N 7 S	100.00	360.0793	-0.6	-0.2	18.7	11.5	even	ok
	5	C 11 H 14 N 5 O 9	58.54	360.0786	-2.5	-2.1	28.1	7.5	even	ok
	6	C 10 H 19 Cl N 3 O 9	56.12	360.0804	2.6	3.3	28.5	2.5	even	ok
	7	C 22 H 15 Cl N O 2	56.01	360.0786	-2.5	-1.7	29.4	15.5	even	ok
	8	C 7 H 11 Cl N 13 O 3	70.10	360.0791	-1.1	-0.7	30.7	8.5	even	ok
	9	C 6 H 15 Cl N 9 O 7	25.99	360.0777	-4.8	-4.2	38.9	3.5	even	ok
	10	C 28 H 10 N	13.87	360.0808	3.6	3.9	68.3	24.5	even	ok
	11	C 20 H 14 N 3 O 2 S	0.57	360.0801	1.8	1.4	140.7	15.5	even	ok
	12	C 5 H 10 N 15 O 3 S	0.05	360.0806	3.2	0.8	171.5	8.5	even	ok

Figure S36. HRMS spectrum of **16.**

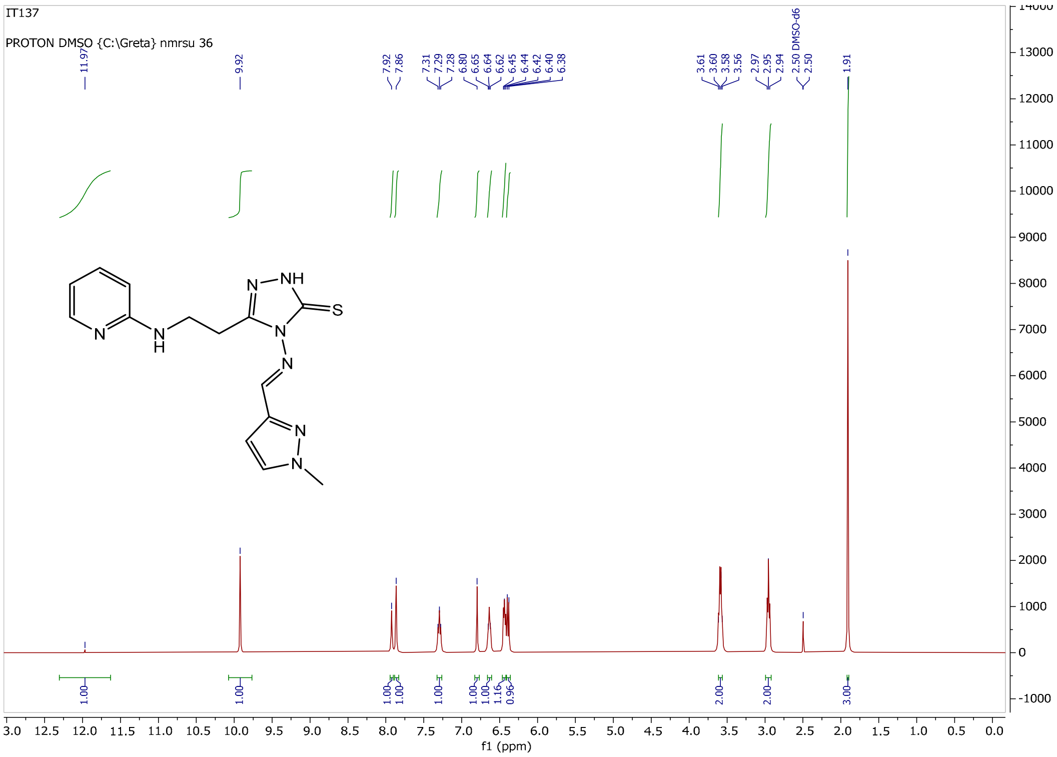

Figure S37. ^{1}H NMR (400 MHz, DMSO-d_6) spectrum of **17**.

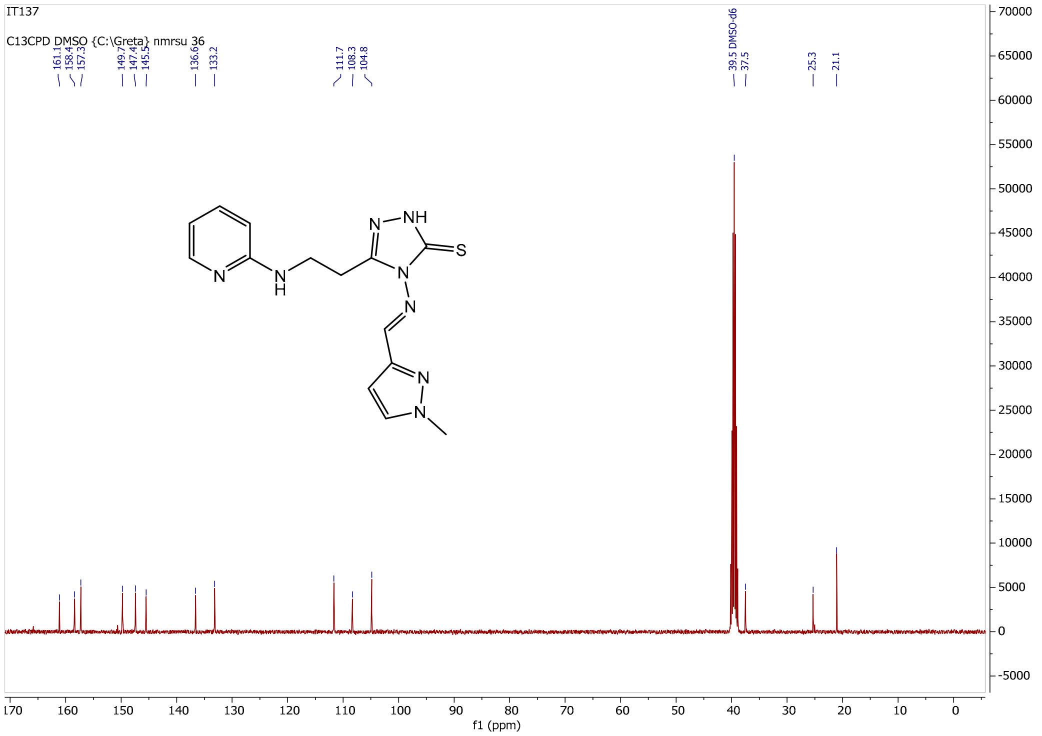

Figure S38. ^{13}C NMR (101 MHz, DMSO-d_6) spectrum of **17.**

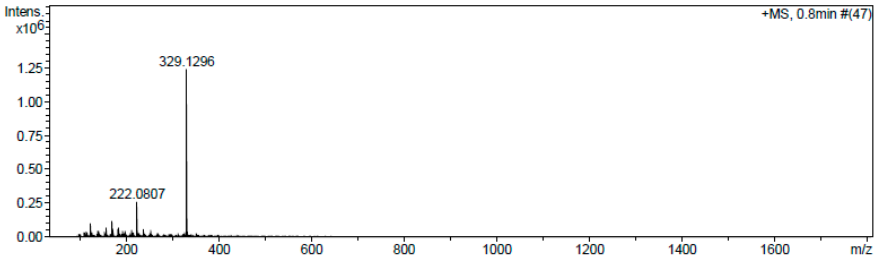

Meas. m/z	#	Formula	Score	m/z	err [ppm]	Mean err [ppm]	mSigma	rdb	e⁻ Conf	N-Rule
329.1296	1	C 13 H 21 N 4 O 4 S	52.97	329.1278	-5.3	-5.0	13.5	5.5	even	ok
	2	C 14 H 17 N 8 S	100.00	329.1291	-1.3	-1.0	20.7	10.5	even	ok
	3	C 9 H 21 N 4 O 9	72.74	329.1303	2.3	2.7	27.4	1.5	even	ok
	4	C 21 H 17 N 2 O 2	32.40	329.1285	-3.3	-2.9	50.2	14.5	even	ok

Figure S39. HRMS spectrum of **17.**

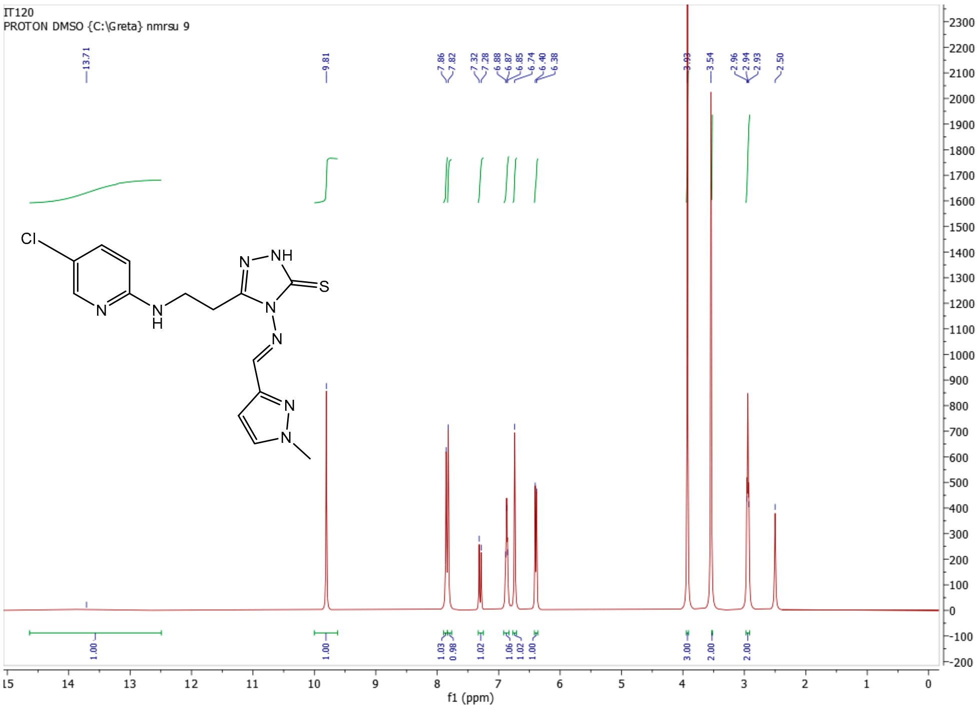

Figure S40. ^{1}H NMR (400 MHz, DMSO-d_6) spectrum of **18** .

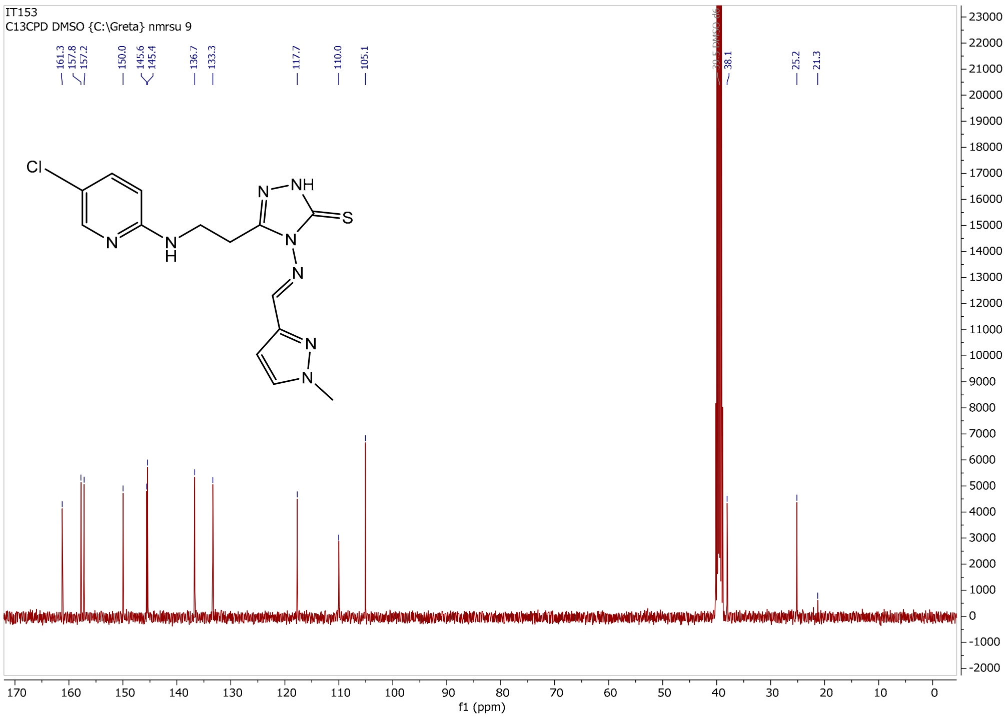

Figure S41. ^{13}C NMR (101 MHz, DMSO-d_6) spectrum of **18.**

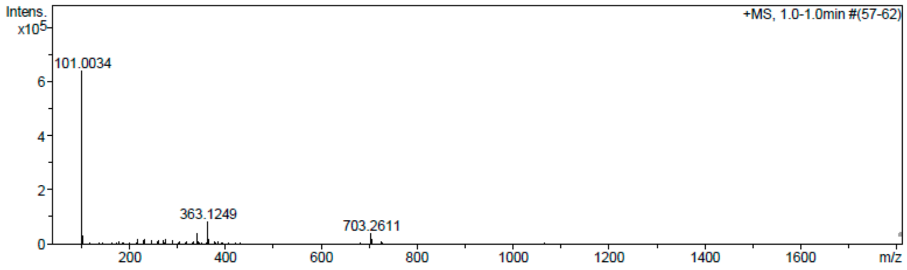

Meas. m/z	#	Formula	Score	m/z	err [ppm]	Mean err [ppm]	mSigma	rdb	e⁻ Conf	N-Rule
341.1428	1	C 18 H 21 N 4 O S	100.00	341.1431	0.6	0.6	7.0	10.5	even	ok
	2	C 17 H 25 O 5 S	54.89	341.1417	-3.3	-3.3	14.1	5.5	even	ok
	3	C 13 H 25 O 10	24.05	341.1442	4.0	5.1	42.2	1.5	even	ok
363.1249	1	C 23 H 15 N 4 O	100.00	363.1240	-2.3	-2.1	35.7	18.5	even	ok
	2	C 15 H 19 N 6 O 3 S	67.56	363.1234	-4.1	-3.6	35.8	9.5	even	ok
	3	C 19 H 23 O 5 S	75.07	363.1261	3.3	3.9	38.6	8.5	even	ok

Figure S42. HRMS spectrum of **18.**

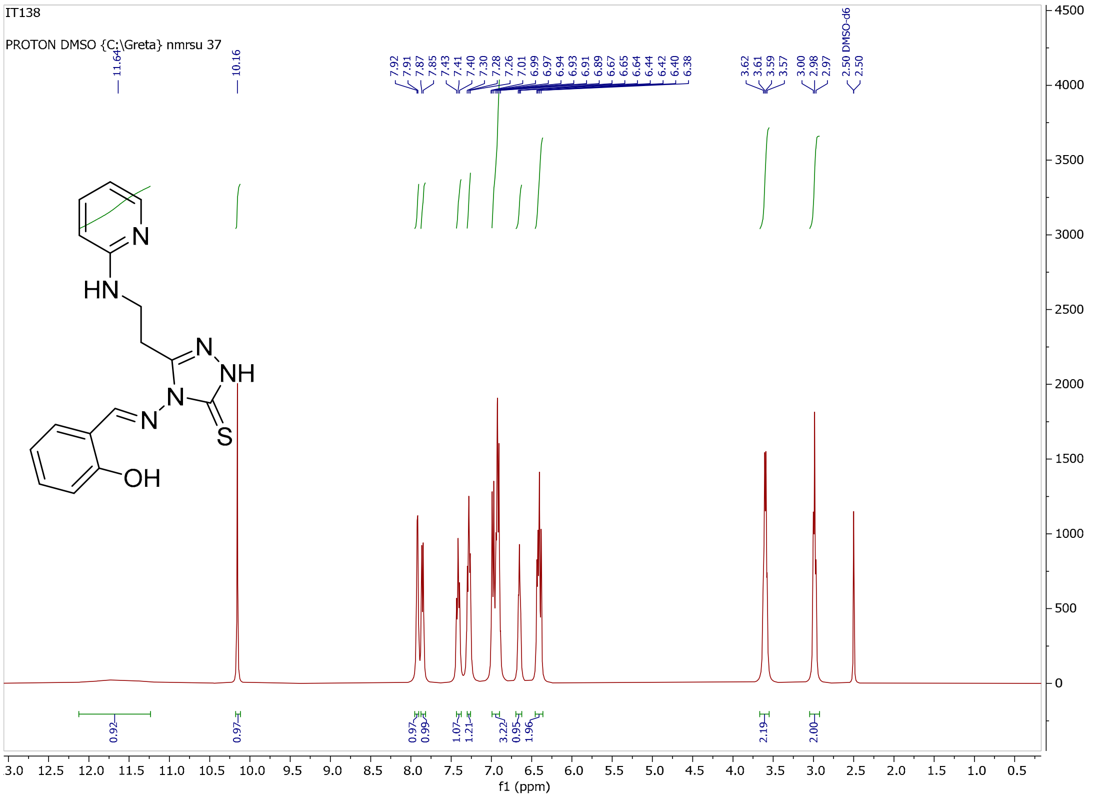

Figure S43. ^{1}H NMR (400 MHz, DMSO-d_6) spectrum of **19**.

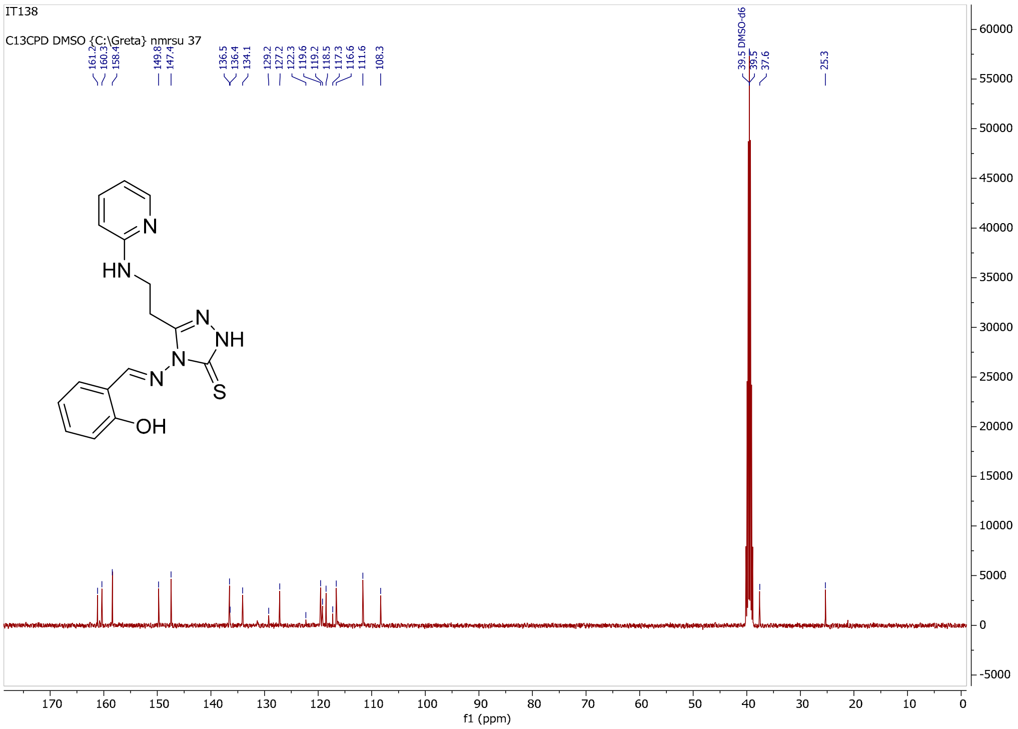

Figure S44. ^{13}C NMR (101 MHz, DMSO-d_6) spectrum of **19.**

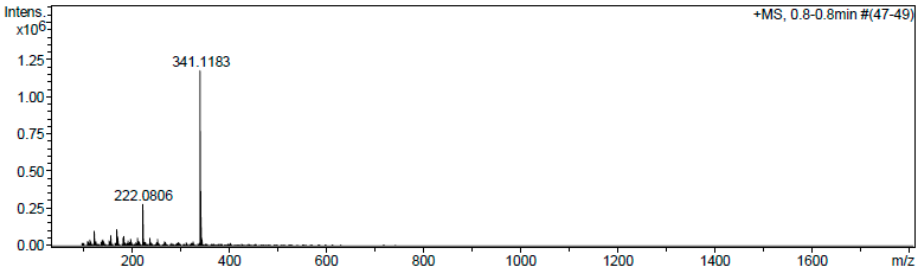

Meas. m/z	#	Formula	Score	m/z	err [ppm]	Mean err [ppm]	mSigma	rdb	e⁻ Conf	N-Rule
341.1183	1	C 15 H 21 N 2 O 5 S	50.89	341.1166	-5.1	-4.8	14.8	6.5	even	ok
	2	C 16 H 17 N 6 O S	100.00	341.1179	-1.1	-0.9	19.8	11.5	even	ok
	3	C 11 H 21 N 2 O 10	73.59	341.1191	2.3	3.4	24.8	2.5	even	ok
	4	C 23 H 17 O 3	32.69	341.1172	-3.1	-2.7	49.3	15.5	even	ok

Figure S45. HRMS spectrum of **19.**

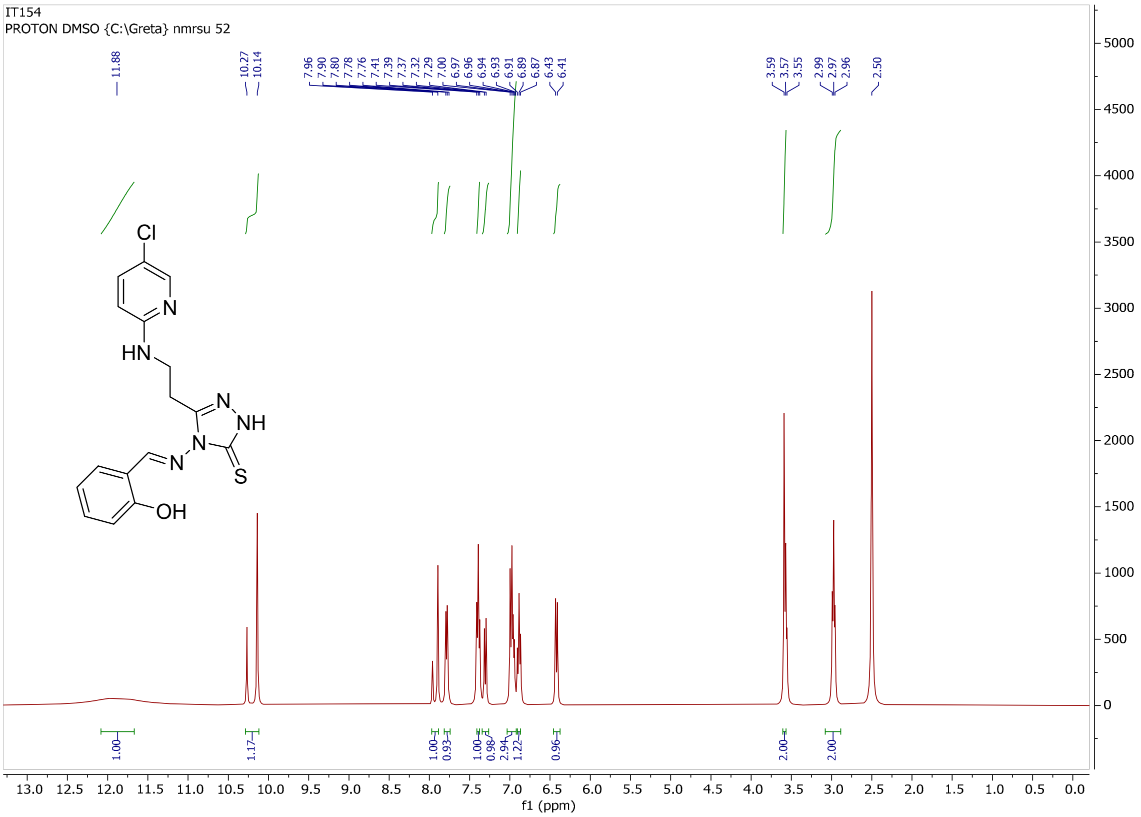

Figure S46. ^{1}H NMR (400 MHz, DMSO-d_6) spectrum of **20**.

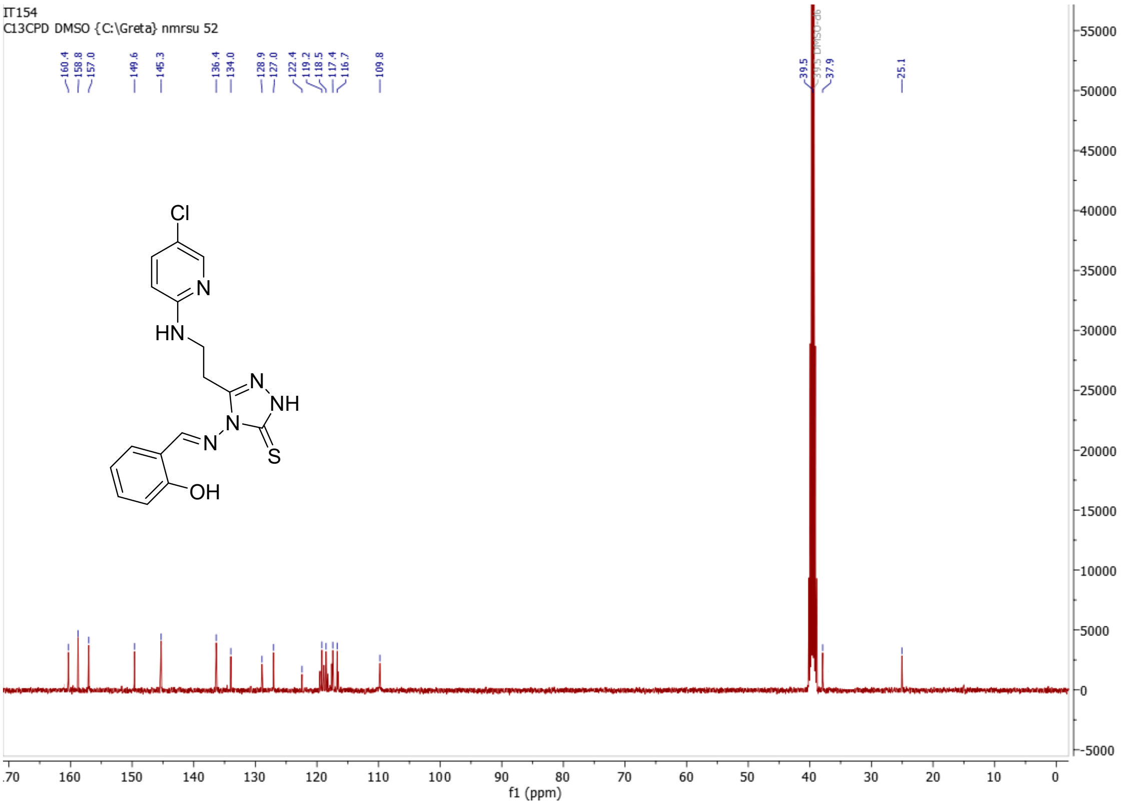

Figure S47. ^{13}C NMR (101 MHz, DMSO-d_6) spectrum of **20.**

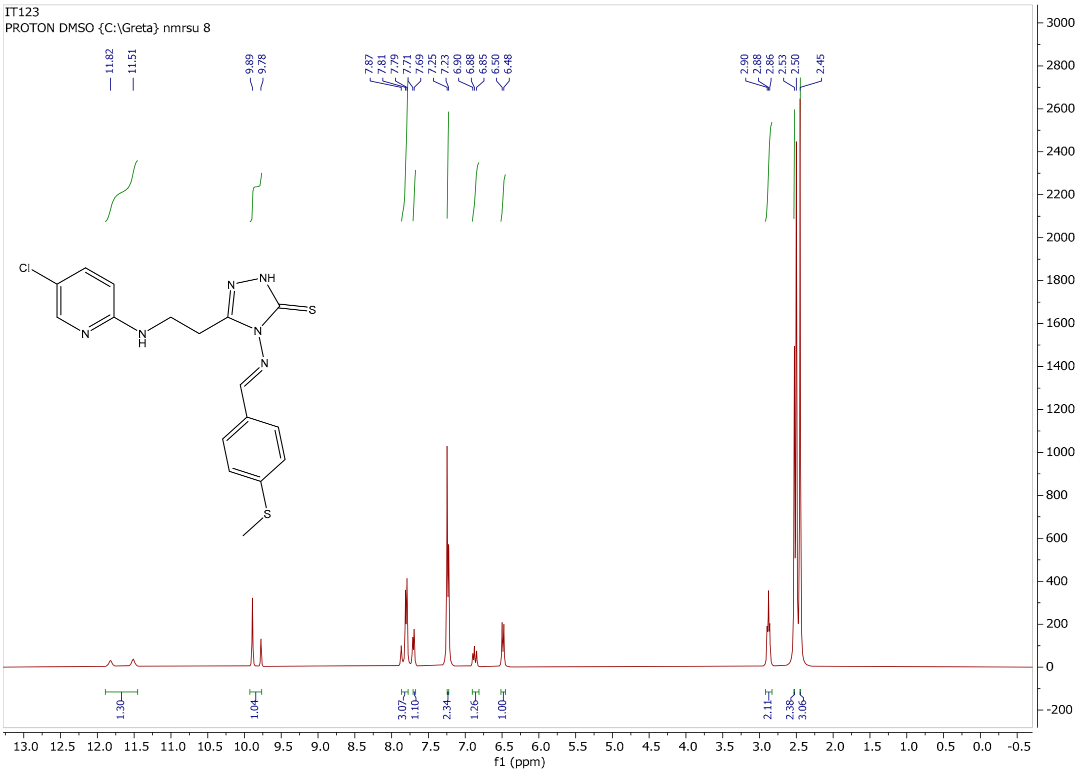

Figure S48. ^{1}H NMR (400 MHz, DMSO-d_6) spectrum of **21** .

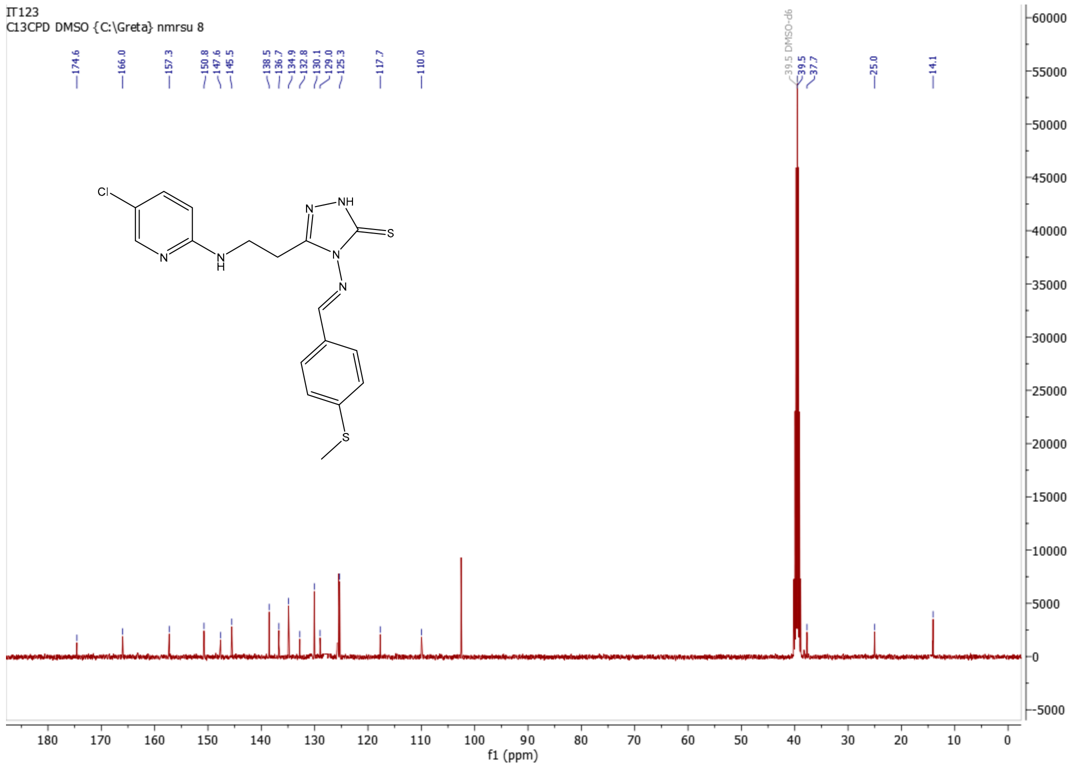

Figure S49. ^{13}C NMR (101 MHz, DMSO-d_6) spectrum of **21.**

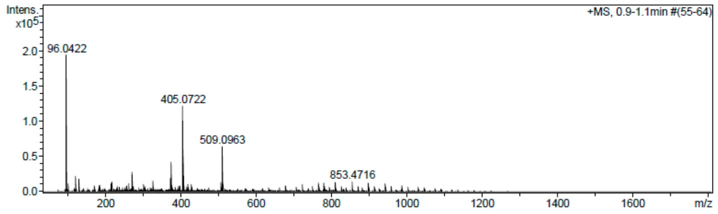

Meas. m/z	#	Formula	Score	m/z	err [ppm]	Mean err [ppm]	mSigma	rdb	e⁻ Conf	N-Rule
405.0722	1	C 16 H 22 Cl N 2 O 4 S 2	46.07	405.0704	-4.5	-4.0	11.2	6.5	even	ok
	2	C 17 H 18 Cl N 6 S 2	100.00	405.0717	-1.2	-0.7	12.7	11.5	even	ok
	3	C 18 H 5 N 12 O	33.37	405.0704	-4.6	-4.2	25.8	22.5	even	ok
	4	C 20 H 18 Cl O 7	47.02	405.0736	3.3	3.9	26.2	11.5	even	ok
	5	C 24 H 18 Cl O 2 S	50.31	405.0711	-2.9	-2.3	27.5	15.5	even	ok
	6	C 21 H 13 N 2 O 7	68.73	405.0717	-1.2	-0.8	29.7	16.5	even	ok
	7	C 16 H 14 Cl N 6 O 5	42.40	405.0709	-3.3	-2.7	29.9	12.5	even	ok
	8	C 12 H 22 Cl N 2 O 9 S	62.29	405.0729	1.7	2.2	30.0	2.5	even	ok
	9	C 9 H 14 Cl N 12 O 3 S	61.51	405.0716	-1.7	-1.2	30.8	8.5	even	ok
	10	C 17 H 10 Cl N 10 O	79.66	405.0722	-0.0	0.5	32.3	17.5	even	ok
	11	C 7 H 5 N 18 O 4	36.72	405.0736	3.3	3.6	36.0	14.5	even	ok
	12	C 22 H 9 N 6 O 3	44.63	405.0731	2.1	2.5	39.7	21.5	even	ok
	13	C 3 H N 24 O 2	26.37	405.0709	-3.3	-5.4	47.7	15.5	even	ok
	14	C 6 H 9 N 14 O 8	47.27	405.0722	0.0	0.3	51.2	9.5	even	ok
	15	C 9 H 17 N 4 O 14	22.06	405.0736	3.3	3.7	53.4	3.5	even	ok
	16	C 5 H 14 Cl N 12 O 8	11.77	405.0741	4.5	5.4	62.5	4.5	even	ok
	17	C 5 H 13 N 10 O 12	15.67	405.0709	-3.3	-2.9	64.0	4.5	even	ok
	18	C 2 H 6 Cl N 22 O 2	19.96	405.0727	1.2	-0.8	70.3	10.5	even	ok
	19	C 30 H 13 S	7.68	405.0732	2.5	2.9	87.2	24.5	even	ok
	20	C 22 H 17 N 2 O 2 S 2	0.41	405.0726	0.9	0.7	147.8	15.5	even	ok
	21	C 7 H 13 N 14 O 3 S 2	0.13	405.0731	2.2	1.8	160.9	8.5	even	ok

Figure S50. HRMS spectrum of **21.**
